# Supplementary material for: CD62L expression level determines the cell fate of myeloid progenitors
Source: Stem Cell Reports. 2021 Nov 18;16(12):2871–86. doi: 10.1016/j.stemcr.2021.10.012 (PMC8693656; doi:10.1016/j.stemcr.2021.10.012)
Supplement: Document S1. Figures S1–S7 [file mmc1.pdf]

Stem Cell Reports, Volume *16*

Supplemental Information

CD62L expression level determines the cell fate of myeloid progenitors

Yusuke Ito, Fumio Nakahara, Yuki Kagoya, and Mineo Kurokawa

Supplemental Figure 1 – CD62L expression reveals the heterogeneity of CMPs in mice and humans

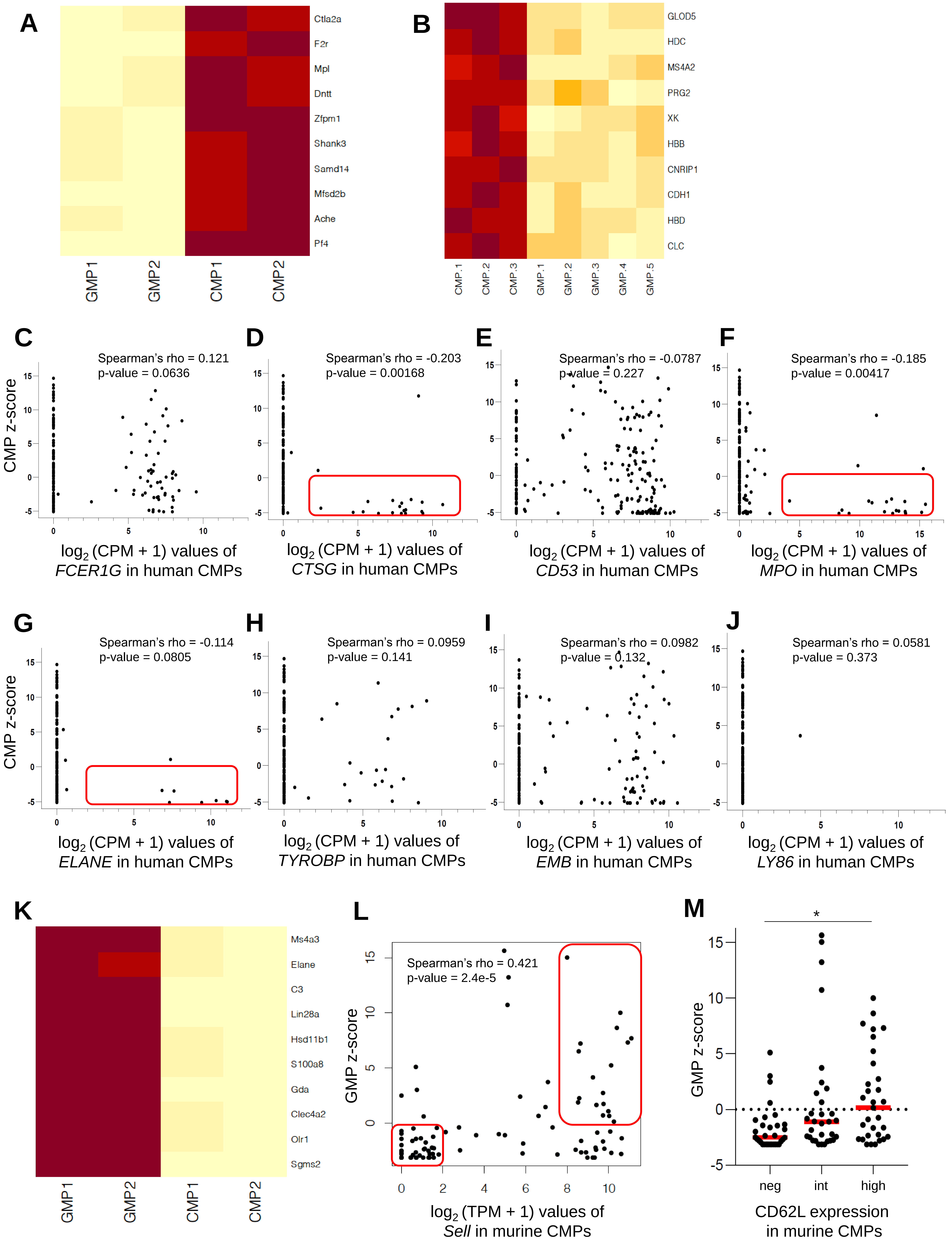

Supplemental Figure 2 – CD62L expression reveals the heterogeneity of CMPs *in vitro*

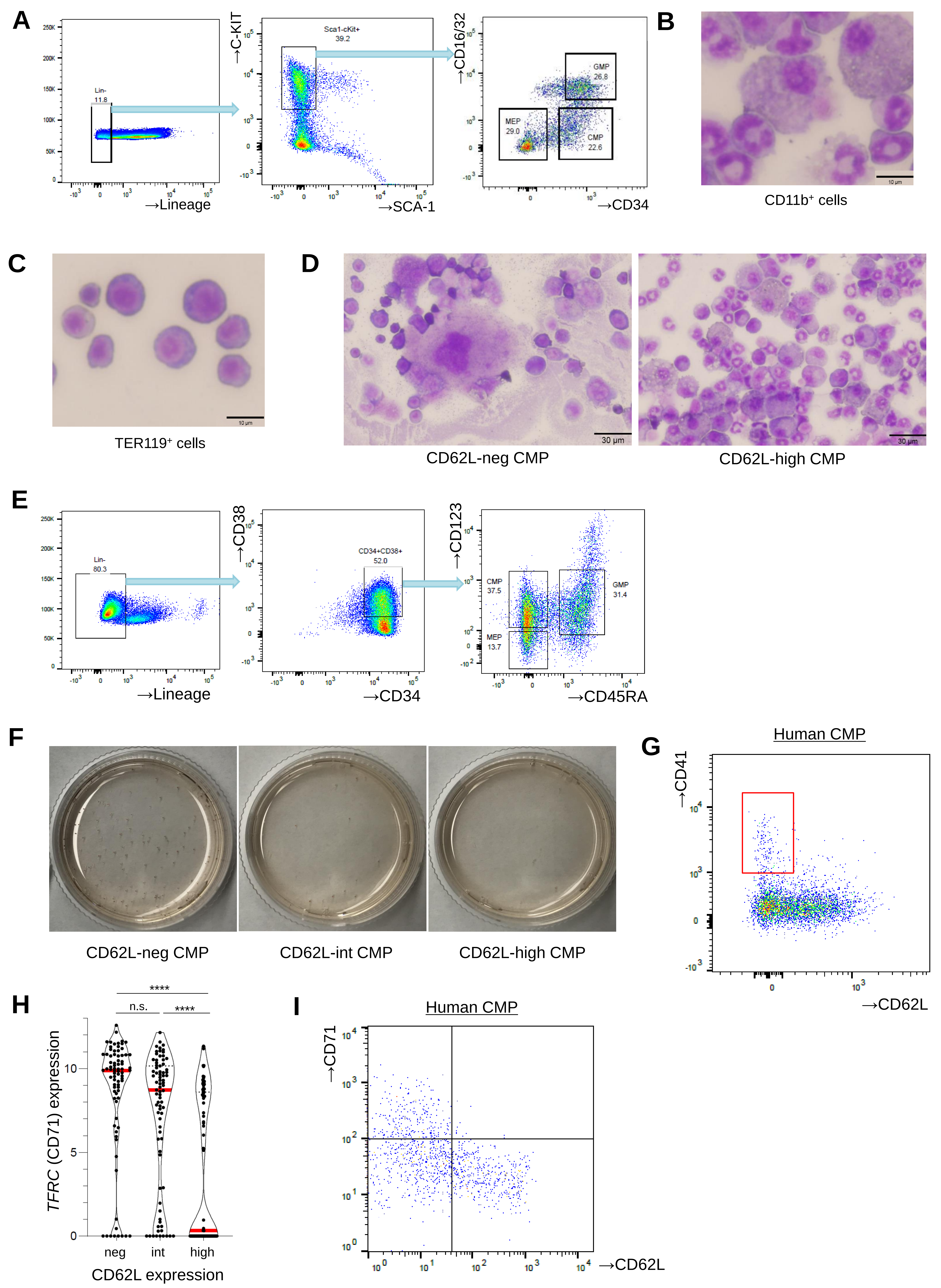

Supplemental Figure 3 – CD62L expression reveals the heterogeneity of CMPs *in vivo*

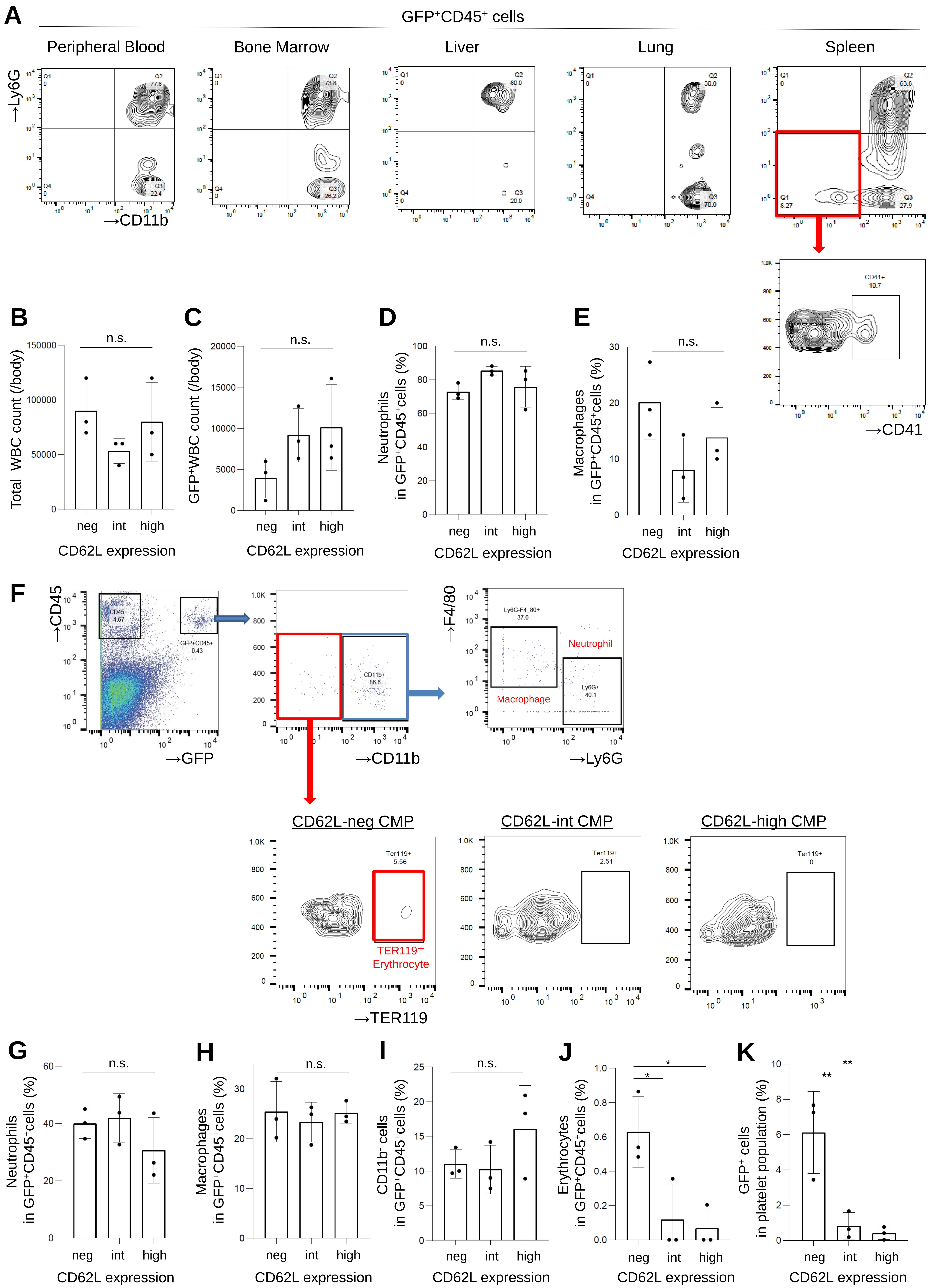

Supplemental Figure 4 – Gene expression profiles of murine CMPs

A

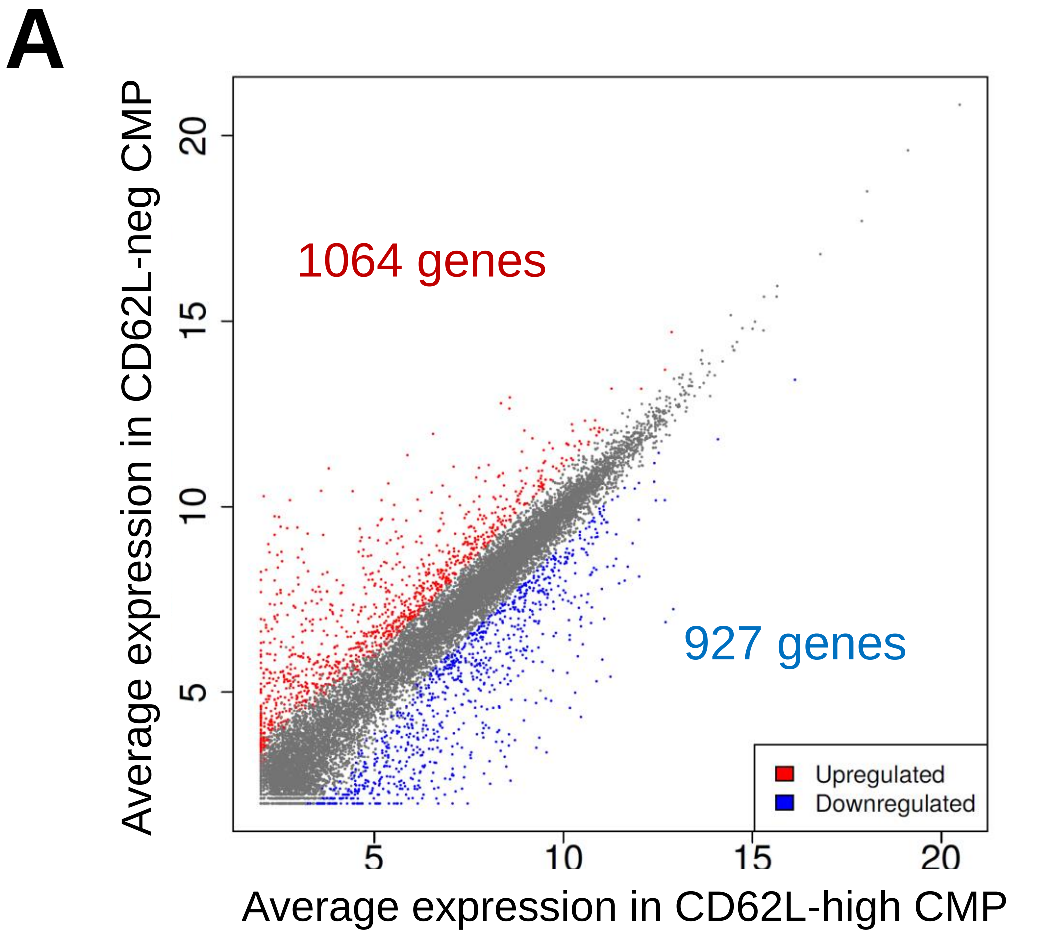

B

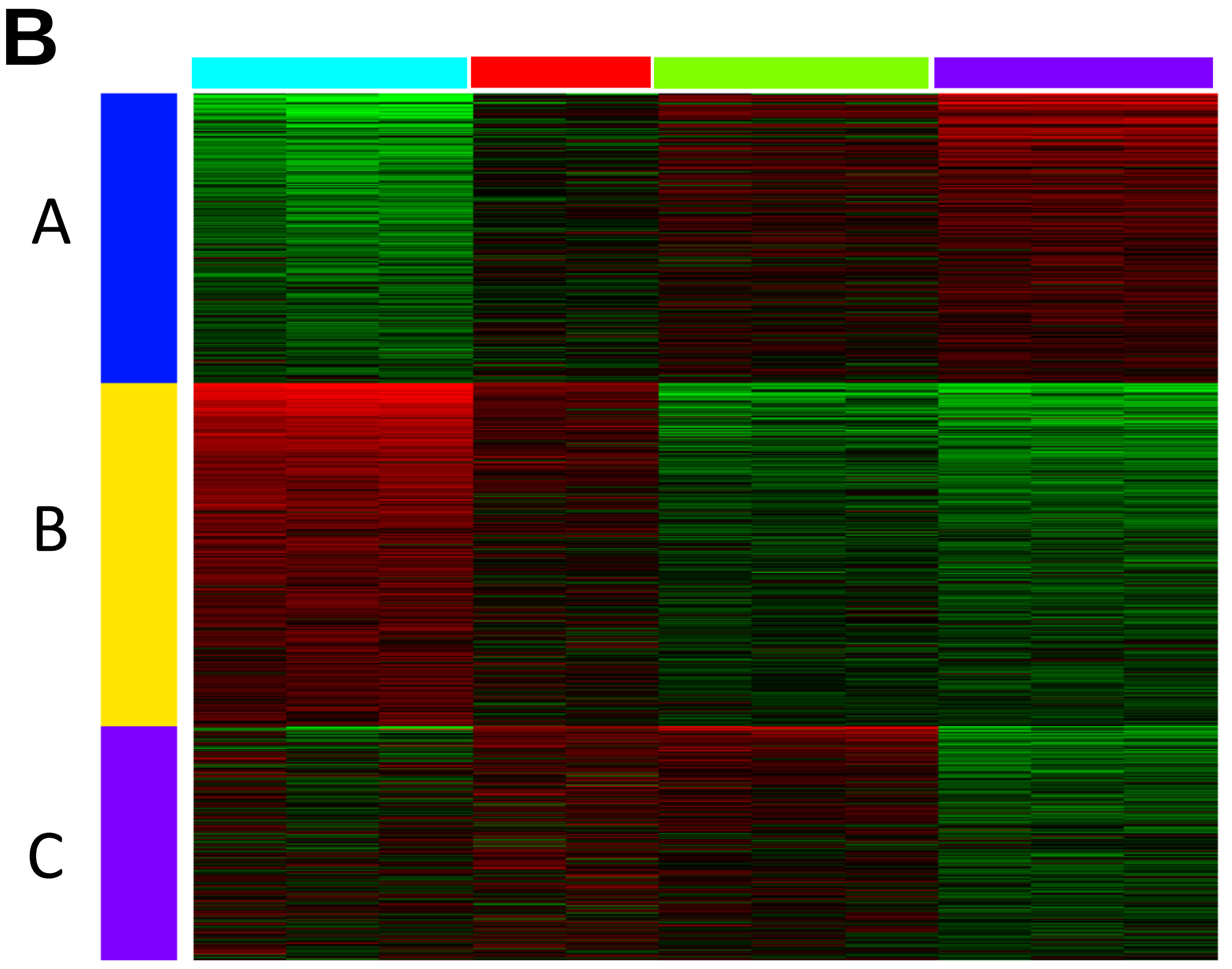

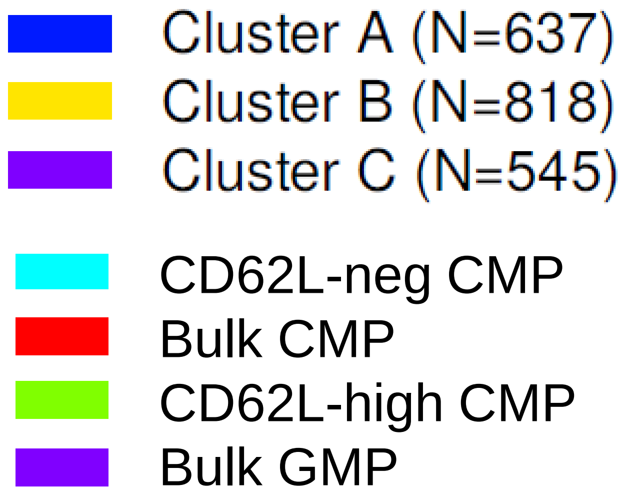

C

	p-value	Enriched Pathways in each cluster
A	1.2e-28	Immune system process
	1.2e-24	Defense response
	1.7e-24	Inflammatory response
	7.2e-24	Immune response
	1.6e-23	Response to external stimulus
	2.6e-21	Cytokine production
B	4.3e-18	Regulation of biological quality
	2.4e-17	Cell adhesion
	2.4e-17	Regulation of multicellular organismal process
	6.4e-16	System development
	7.4e-16	Regulation of response to stimulus
	2.6e-15	Blood coagulation
C	8.8e-08	Cell activation
	8.8e-08	Leukocyte activation
	1.4e-07	Positive regulation of immune system process
	2.5e-07	Cell surface receptor signaling pathway
	3.4e-07	Cell adhesion
	3.4e-07	Positive regulation of cell activation

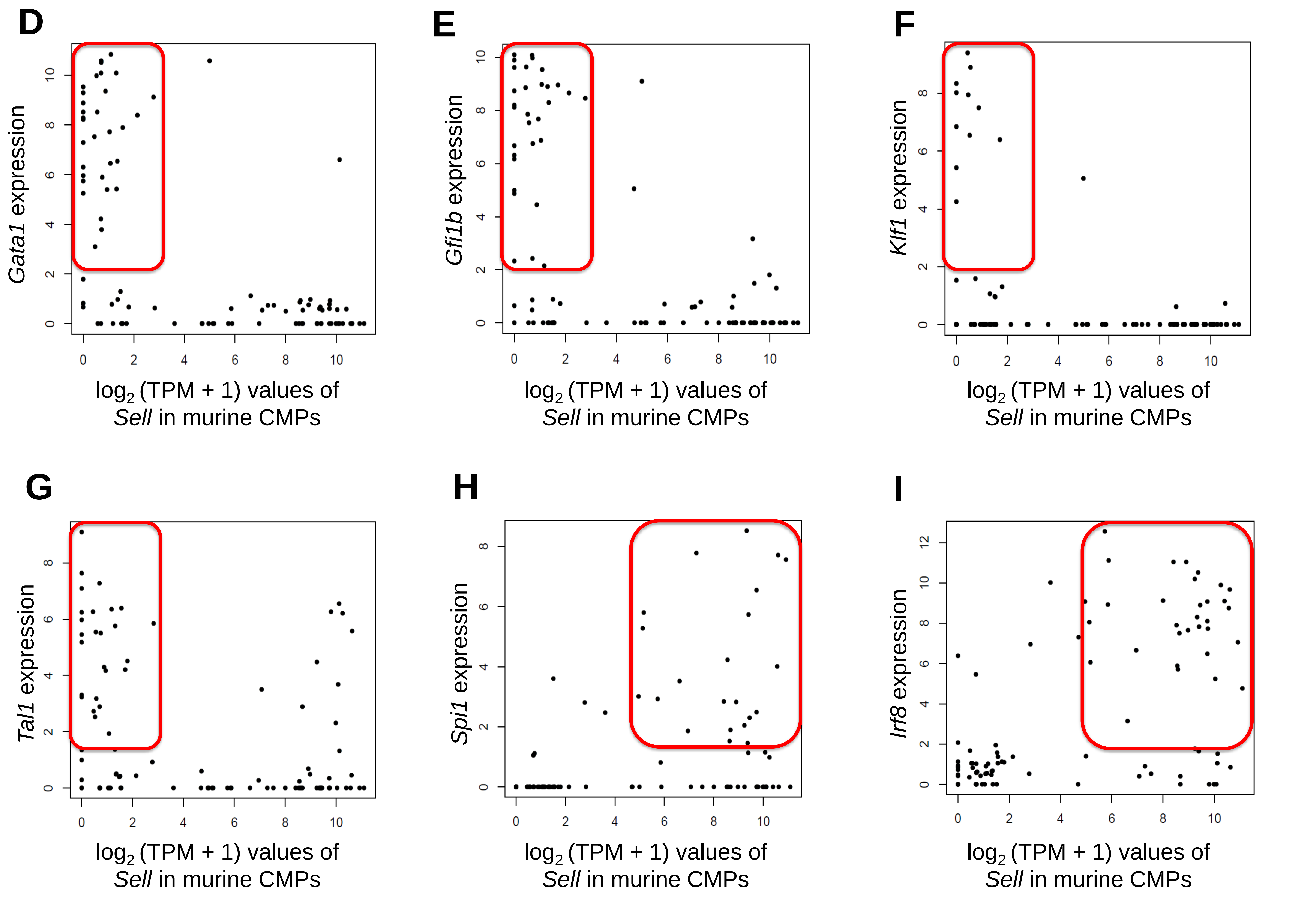

Supplemental Figure 5 – Single cell analyses confirm the heterogeneity of CMPs and GMPs

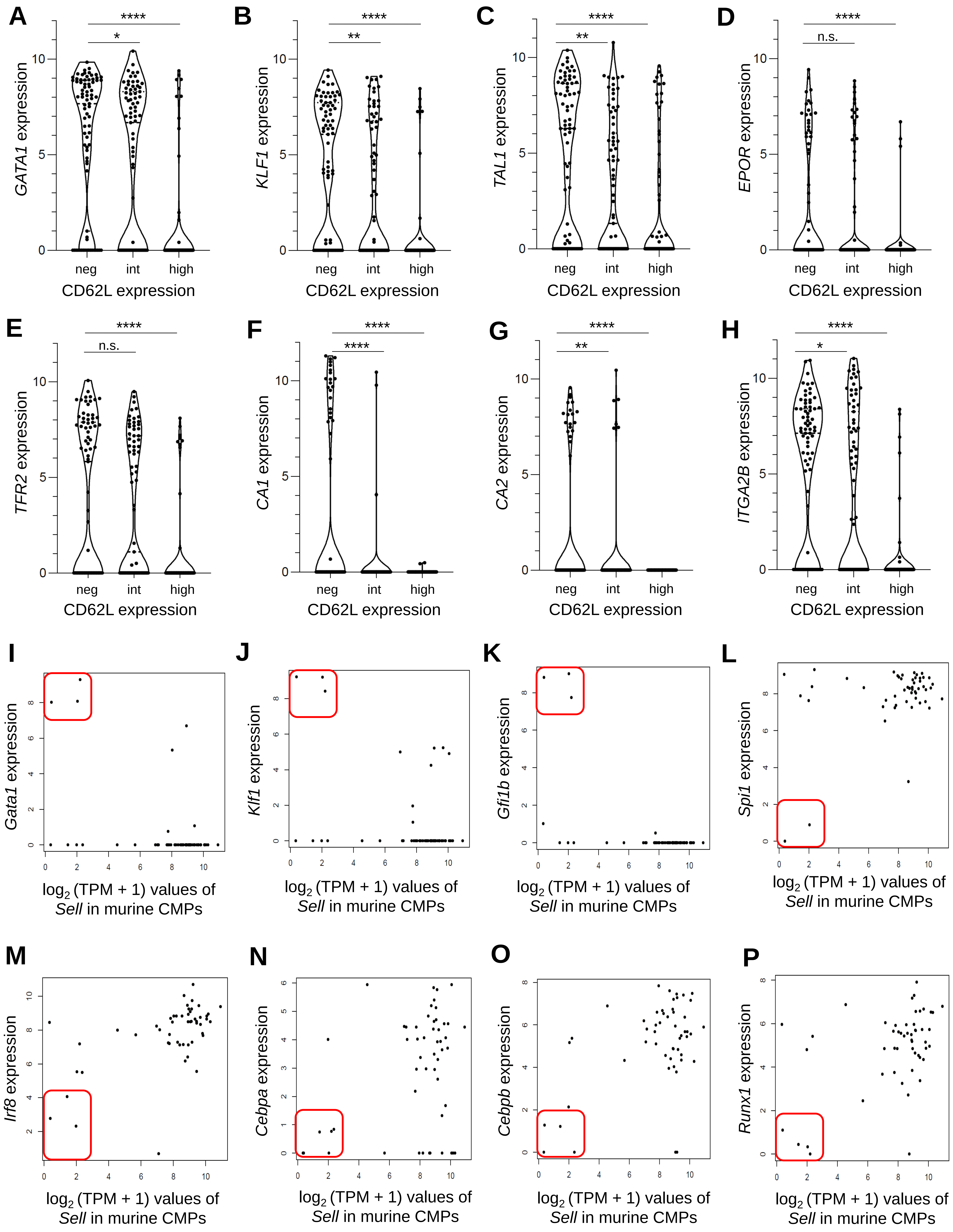

Supplemental Figure 6 – CD62L expression reveals the heterogeneity of GMPs in mice

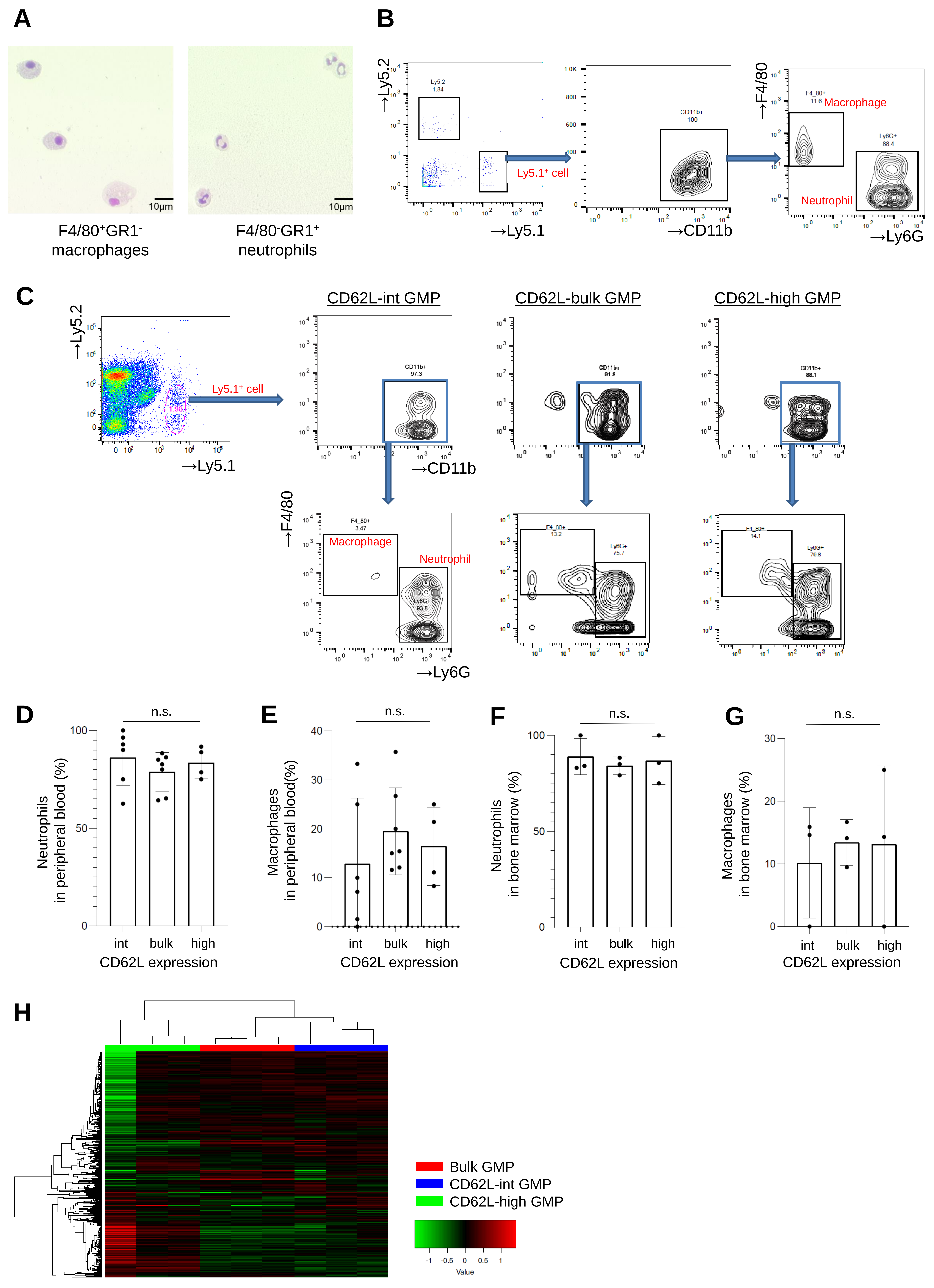

Supplemental Figure 7 – The summary of murine myeloid differentiation

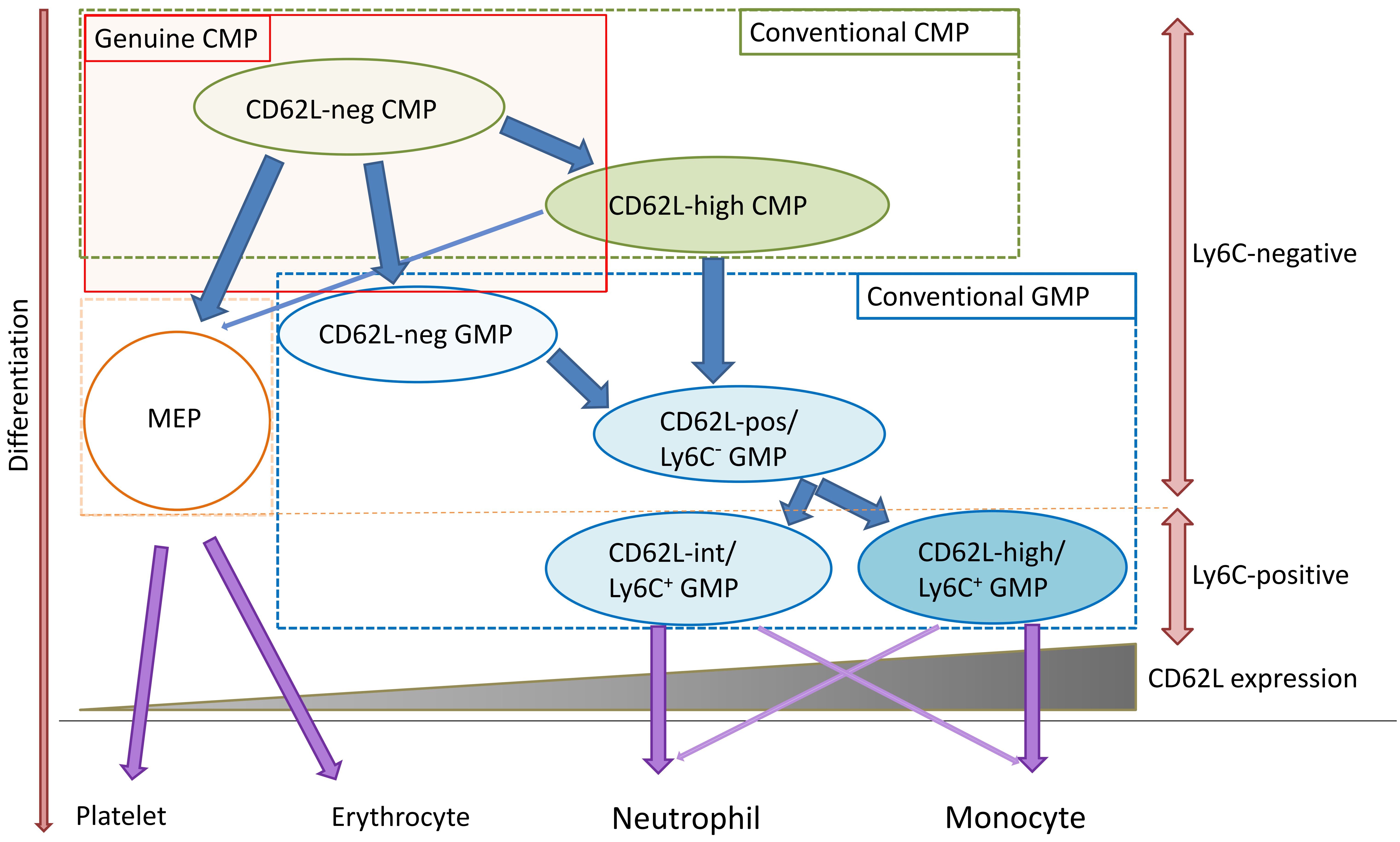

Supplemental Information

Supplemental Table legends

Table S1. Top 200 CMP and GMP signature gene sets in mice, related to Figure 4 and 6.

Murine CMP signature gene set and GMP signature gene set.

Supplemental Figure legends

Figure S1. CD62L expression reveals the heterogeneity of CMPs in mice and humans, related to Figure 1. (A and B) Heatmap of ten genes which were most highly expressed in CMPs compared with GMPs, using bulk RNA-seq data in mice (GSE116177) (A) and humans (GSE42519) (B). (C-J) Expression levels of each gene were plotted against the sum of CMP z-scores in individual cells in humans (C: *FCER1G*, D: *CTSG*, E: *CD53*, F: *MPO*, G: *ELANE*, H: *TYROBP*, I: *EMB*, J; *LY86*). X-axis shows $\log_2$ (CPM + 1) value of each gene and y-axis shows the sum of z-scores. (K) Heatmap of ten genes which were most highly expressed in murine GMPs compared with CMPs, using bulk RNA-seq data (GSE116177). (L) Expression levels of *Sell* were plotted against the sum of murine GMP z-scores in individual cells. X-axis shows $\log_2$ (TPM + 1) value of *Sell* and y-axis shows the sum of z-scores. (M) The plot

of the GMP z-scores divided by CD62L-neg, int, and high CMPs in mice. Statistical significance was calculated using one-way ANOVA. (*$p < 0.05$).

Figure S2. CD62L expression reveals the heterogeneity of CMPs *in vitro*, related to Figure 2. (A) Representative flow cytometry plots for sorting murine progenitors (CMP, GMP, and MEP) within mouse bone marrow cells. (B-D) Wright-Giemsa staining of cells after 7-day culture in semisolid medium. (B) $CD11b^+$ cells, (C) $TER119^+$ cells, (D) colonies derived from CD62L-neg and high GMPs. (E) Representative flow cytometry plots for sorting human progenitors (CMP, GMP, and MEP) within human $CD34^+$ bone marrow cells. (F) Macroscopic findings of colony-forming cell assay of human CMPs. CD62L-neg CMPs generate more red-colored BFU-Es than others. Experiments were performed three times. (G) Representative flow cytometry plots of CD41 and CD62L co-staining of human CMPs. Experiments were performed three times. (H) Violin plot of *TFRC*, a gene encoding CD71, in human CMPs using single cell RNA-seq data (GSE113046). CMPs were divided into CD62L-neg, int, and high CMPs, and Y-axis shows $\log_2$ (CPM + 1) of *TFRC* expression. Statistical significance was calculated using one-way ANOVA. ****$p < 0.0001$, n.s.: not significant. (I) Representative flow cytometry plots of CD71 and CD62L co-staining of human CMPs. Experiments were

performed three times.

Figure S3. CD62L expression reveals the heterogeneity of CMPs *in vivo*, related to Figure 3. (A) Representative flow cytometry plots of CD11b, Ly6G, and CD41 staining of GFP-positive hematopoietic cells collected from peripheral blood, bone marrow, liver, lung, and spleen in mice transplanted with CD62L-neg CMPs derived from GFP-mice. Experiments were performed three times. (B-E) The result of (B) total $CD45^+$ cell number, (C) GFP-positive cell number, and the proportion of (D) neutrophils and (E) macrophages in GFP-positive cells collected from bone marrow. Means ± S.D. of three independent experiments. Statistical significance was calculated using one-way ANOVA. (n.s.: not significant, n = 3). (F) Representative flow cytometry plots of neutrophils, macrophages, and erythrocytes in GFP-positive hematopoietic cells collected from splenic cells. Experiments were performed three times. (G-K) The result of the proportion of (G) neutrophils, (H) macrophages, (I) $CD11b^-$ cells, and (J) erythrocytes in GFP-positive hematopoietic cells and (K) the proportion of GFP-positive platelets in total platelets collected from spleen. Means ± S.D. of three independent experiments. Statistical significance was calculated using one-way ANOVA. (*$p < 0.05$, **$p < 0.01$, n.s.: not significant, n = 3).

Figure S4. Gene expression profiles of murine CMPs, related to Figure 4. (A) The scatter plot of differentially expressed genes between CD62L-neg CMPs and CD62L-high CMPs. Fold change > 2, FDR < 0.05. (B) The result of k-means clustering of bulk CMPs, CD62L-neg CMPs, CD62L-high CMPs, and bulk GMPs divided into cluster A, B, and C. (C) The result of pathway enrichment analysis of each cluster, showing top 6 significantly enriched pathways. (D-I) The relationship between several transcription factors and *Sell* expression in murine CMPs at single cell level (GSE70236). X-axis shows $\log_2$ (TPM + 1) of *Sell* and y-axis shows $\log_2$ (TPM + 1) of each transcription factor (D: *Gata1*, E: *Gfi1b*, F: *Klf1*, G: *Tal1*, H: *Spi1*, I: *Irf8*).

Figure S5. Single cell analyses confirm the heterogeneity of CMPs and GMPs, related to Figure 4 and 6. (A-H) Violin plots of several genes in human CMPs using single cell RNA-seq data (GSE113046). CMPs were divided into CD62L-neg, int, and high CMPs, and Y-axis shows $\log_2$ (CPM + 1) of each gene (A: *GATA1*, B: *KLF1*, C: *TAL1*, D: *EPOR*, E: *TFR2*, F: *CA1*, G: *CA2*, H: *ITGA2B* (CD41)). Statistical significance was calculated using one-way ANOVA. *$p < 0.05$, **$p < 0.01$, ****$p < 0.0001$, n.s.: not significant. (I-P) The relationship between several transcription factors

and CD62L expression in murine GMPs at single cell level (GSE130966). X-axis shows $\log_2$ (TPM + 1) of *Sell* and y-axis shows each transcription factor (I: *Gata1*, J: *Klf1*, K: *Gfi1b*, L: *Spi1*, M: *Irf8*, N: *Cebpa*, O: *Cebpb*, P: *Runx1*).

Figure S6. CD62L expression reveals the heterogeneity of GMPs in mice, related to Figure 7. (A) May-Giemsa staining of F4/80$^+$ macrophages and GR-1$^+$ neutrophils after 7-day culture of murine GMPs. (B and C) Representative plots of neutrophils and macrophages in the Ly5.1-positive donor cell population in (B) peripheral blood and (C) spleen are shown. (D-G) The result of the proportion of (D) neutrophils and (E) macrophages in peripheral blood, and (F) neutrophils and (G) macrophages in bone marrow in Ly5.1-positive donor cell population. Means ± S.D. of three to six independent experiments. Statistical significance was calculated using one-way ANOVA. (n.s.: not significant). (H) The result of hierarchical clustering of CD62L-int GMPs, CD62L-high GMPs, and bulk GMPs.

Figure S7. The summary of murine myeloid differentiation

Proposed model of CMP and GMP differentiation in mice. CD62L-neg CMPs are genuine CMPs and located at the upper differentiation hierarchy, whereas CD62L-high

CMPs were highly skewed to GMP potential. CD62L-negative GMPs are the most immature subsets, and differentiate into CD62L-positive GMPs in one direction. Then, Ly6C expression becomes upregulated and Ly6C^{+}/CD62L-int GMPs are skewed to neutrophil differentiation, whereas Ly6C^{+}/CD62-high GMPs are skewed to monocyte differentiation.
