# Supplementary material for: CD62L expression level determines the cell fate of myeloid progenitors
Source: Stem Cell Reports. 2021 Nov 18;16(12):2871–86. doi: 10.1016/j.stemcr.2021.10.012 (PMC8693656; doi:10.1016/j.stemcr.2021.10.012)
Supplement: Document S2. Article plus supplemental information [file mmc3.pdf]

Stem Cell Reports
Article
OPEN ACCESS

CD62L expression level determines the cell fate of myeloid progenitors

Yusuke Ito,[1,2] Fumio Nakahara,[1] Yuki Kagoya,[3] and Mineo Kurokawa[1,3,*]
[1]Department of Hematology and Oncology, Graduate School of Medicine, The University of Tokyo, Bunkyo-ku, Tokyo 113-8655, Japan
[2]Research Fellow of Japan Society for the Promotion of Science, Tokyo, Japan
[3]Department of Cell Therapy and Transplantation Medicine, The University of Tokyo Hospital, Bunkyo-ku, Tokyo, 113-8655, Japan
*Correspondence: kurokawa@m.u-tokyo.ac.jp
https://doi.org/10.1016/j.stemcr.2021.10.012

SUMMARY

Hematopoietic cells differentiate through several progenitors in a hierarchical manner, and recent single-cell analyses have revealed substantial heterogeneity within each progenitor. Although common myeloid progenitors (CMPs) are defined as a multipotent cell population that can differentiate into granulocyte-monocyte progenitors (GMPs) and megakaryocyte-erythrocyte progenitors (MEPs), and GMPs generate neutrophils and monocytes, these myeloid progenitors must contain some lineage-committed progenitors. Through gene expression analysis at single-cell levels, we identified CD62L as a marker to reveal the heterogeneity. We confirmed that CD62L-negative CMPs represent "bona fide" CMPs, whereas CD62L-high CMPs are mostly restricted to GMP potentials both in mice and humans. In addition, we identified CD62L-negative GMPs as the most immature subsets in GMPs and Ly6C^{+}CD62L-intermediate and Ly6C^{+}CD62L-high GMPs are skewed to neutrophil and monocyte differentiation in mice, respectively. Our findings contribute to more profound understanding about the mechanism of myeloid differentiation.

INTRODUCTION

Hematopoietic cells differentiate from hematopoietic stem and progenitor cells in a strictly regulated hierarchical manner to maintain homeostasis (Akashi et al., 2000). Multipotent hematopoietic stem cells differentiate into committed progenitor cells with differentiation capacity into more restricted lineages. Although the differentiation status is mainly discriminated by surface marker profiles, recent studies using single-cell analyses and lineage-tracing approaches have revealed the heterogeneity and lineage skewing in hematopoietic stem and progenitor cells (Buenrostro et al., 2018; Dinh et al., 2020; Drissen et al., 2019; Jacobsen and Nerlov, 2019; Kwok et al., 2020; Nestorowa et al., 2016; Olsson et al., 2016; Paul et al., 2015; Weinreb et al., 2020). These reports have challenged the classical hierarchical model of hematopoiesis and provided a revised framework indicating a continuum of differentiation (Laurenti and Göttgens, 2018; Loughran et al., 2020; Notta et al., 2016; Velten et al., 2017). Scrutinizing the heterogeneity in the progenitor population has clarified the difference in expression of genes important for differentiation between each subpopulation, which contributes to elucidating the hematopoietic differentiation mechanism more minutely (Yáñez et al., 2015).

With regard to myeloid progenitors, several articles have investigated the heterogeneity of common myeloid progenitors (CMPs) (Miyawaki et al., 2017; Mori et al., 2015; Nishikii et al., 2015) and granulocyte-monocyte progenitors (GMPs) (Dinh et al., 2020; Kawamura et al., 2017; Kwok et al., 2020; Yáñez et al., 2015), which have revealed the existence of lineage-committed subgroups in these progenitors. Moreover, Notta et al. (2016) suggest that the stem cell compartment is multipotent but that the progenitors are unipotent in adult human bone marrow. These previous reports suggest that CMPs contain the GMP-skewed subset, but the specific surface marker identifying this population has not been clarified.

Traditional surface markers defining CMPs and GMPs are quite different between mice and humans. Human CMPs are defined as Lineage^{-}CD34^{+}CD38^{+}CD45RA^{-}CD123^{mid}, and GMPs as Lineage^{-}CD34^{+}CD38^{+}CD45RA^{+}CD123^{mid} (Manz et al., 2002), whereas murine CMPs are defined as Lineage^{-}SCA-1^{-}C-KIT^{+}CD16/32^{-}CD34^{+}, and murine GMPs as Lineage^{-}SCA-1^{-}C-KIT^{+}CD16/32^{+}CD34^{+} (Akashi et al., 2000). Identification of common molecular profiles between the human and mouse will make it easier to translate the findings obtained in mouse studies to humans.

In this study, we analyzed heterogeneity of human and murine CMPs using single-cell RNA sequencing (RNA-seq) data and identified CD62L as a useful marker to clarify functional heterogeneity of the myeloid progenitor population in both mice and humans. These findings elucidate the myeloid cell differentiation diagram in more detail.

RESULTS

Differential CD62L expression levels reveal heterogeneity within the CMP population in mice and humans

To elucidate heterogeneity of myeloid progenitors, we analyzed gene expression profiles of individual cells within the human and mouse CMP populations using publicly available single-cell RNA-seq data (GSE70236 and GSE113046) (Drissen et al., 2019; Olsson et al., 2016). To

This is an open access article under the CC BY license (http://creativecommons.org/licenses/by/4.0/).

extract the gene sets that represent the CMP attributes, we first selected the top 10 genes that were most highly expressed in murine CMPs compared with the downstream population, GMPs, using bulk RNA-seq data (GSE116177) (Choi et al., 2019) (Figure S1A). When analyzed at single-cell levels, the expression signatures of the CMP genes showed substantial heterogeneity (Figure 1A). We hypothesized that the cells with low CMP scores are more differentiated toward GMP. To find molecular profiles that mark the differentiated population within CMPs, we compared gene expression data between cells with high and low expression of CMP genes and extracted the top 10 genes highly expressed in cells with low CMP scores (Figure 1B). To explore genes whose expression patterns were similar in human cells, we calculated the CMP score using human transcriptome data (GSE42519) (Rapin et al., 2014), which also showed substantial heterogeneity (Figures 1C and S1B). Analysis of the expression of the ten candidate genes in Figure 1B showed that the expression of *CTSG*, *MPO*, and *ELANE* was restricted to CMPs with low CMP scores, all of which are well known as neutrophil/monocyte-specific genes (Olsson et al., 2016), suggesting that CMPs with low CMP signature scores are more differentiated into neutrophil/monocyte-lineage cells (Figures 1D, S1C, and S1J). Among the surface molecule-encoding genes, *SELL*, the gene encoding CD62L, was differentially expressed between cells with the high and low CMP signature score in both the human and mouse CMP (Figures 1D–1G). Likewise, we extracted GMP signature gene sets (Figure S1K), and *Z* score calculation showed that CD62L-high CMPs expressed GMP-specific genes compared with CD62L-negative CMPs (Figures S1L and S1M). Based on these results, we focused on CD62L as a promising molecule whose expression explains heterogeneity of the myeloid progenitor population, suggesting that CD62L-negative CMPs represent "bona fide" CMPs, and that CD62L-high CMPs are skewed to GMP potential.

Differential CD62L expression segregates differentiation potential of the CMP

Consistent with heterogeneity at gene expression levels, CD62L expression at protein levels was widely distributed in murine CMPs as well as GMPs, while it was almost negative in MEPs (Figures 2A and S2A). CMPs were trisected according to CD62L expression level and defined as CD62L-negative (CD62L-neg), CD62L-intermediate (CD62L-int), and high CMPs (Figure 2A). Colony-forming cell assays of CD62L-neg, CD62L-int, and high CMPs revealed that CD62L-neg CMPs produced BFU-E (19.9% ± 3.5%), CFU-Meg (8.1% ± 1.3%), and CFU-GEMM (10.7% ± 1.9%). On the other hand, CD62L-int and CD62L-high CMPs were skewed into granulocyte and monocyte colonies (CD62L-int CMPs: 96.7% ± 3.2%, CD62L-high CMPs: 99.4% ± 0.8%) (Figure 2B). Consistent with this, most of the cells within the colonies derived from CD62L-high CMPs were CD11b positive, while a part of the cells from CD62L-neg CMPs was positive for the erythrocyte marker TER119 (Figures 2C–2E, S2B, and S2C). Giemsa staining showed that colonies derived from CD62L-high CMPs were mostly terminally differentiated into macrophages or neutrophils, whereas colonies from CD62L-neg CMPs contained immature myeloid cells, megakaryoblasts, and erythroblasts (Figure S2D). When incubated with the liquid medium, CD62L-neg CMPs generated both GMPs (45.0% ± 3.9%) and MEPs (16.9% ± 1.1%), while CD62L-high CMPs mostly produced GMPs (75.8% ± 10.0%) and the frequency of cells with an MEP phenotype were only 1.5% ± 0.9% (Figure 2F). Also, while CD62L-neg CMPs differentiated into CD62L-high CMPs, CD62L-high CMPs did not produce CD62L-neg cells, suggesting that CD62L is upregulated along with the differentiation of CMPs (Figure 2G).

Next, we examined the expression patterns of CD62L in human CMPs to investigate whether these findings are recapitulated in humans. As was seen in murine cells, the expression of CD62L was widely distributed within the human CMP population (Figures 2H and S2E). Colony-forming cell assay of CD62L-neg, CD62L-int, and high CMPs demonstrated that CD62L-neg CMPs were skewed to generate BFU-E (71.5% ± 3.4%), whereas CD62L-high CMPs mostly generated CFU-G/M/GM (87.0% ± 0.9%) (Figure 2I). Macroscopically, human BFU-Es were detected as red-colored colonies (Figure S2F). Cells differentiated from CD62L-neg CMPs were almost positive for CD235a (Figure 2J). Previous studies have shown that CD41-positve CMPs are megakaryocyte-specific progenitors in humans (Miyawaki et al., 2017). When human CMPs were analyzed for CD41 and CD62L expression, most CD41-positive CMPs were CD62L-negative, suggesting that CMPs that are capable of differentiating into megakaryocytes are confined to the CD62L-neg population (Figure S2G). Another study has shown that CD71-positive CMPs are erythrocyte specific (Notta et al., 2016). The expressions of CD71 and CD62L in CMPs were mutually exclusive (Figures S2H and S2I). Collectively, these data suggest that CD62L-neg CMPs are genuine CMPs that can differentiate into myeloid as well as erythro-megakaryocytic cells, whereas CD62L-high CMPs are skewed to GMP potential *in vitro* both in mice and humans.

CD62L-neg but not CD62L-high CMP differentiates into megakaryocytic cells *in vivo*

To further elucidate the difference between CD62L-neg and CD62L-high CMPs, we focused on the capacity to produce platelets. When murine CMPs were cultured in liquid medium, microscopic imaging showed that megakaryocytes with large cytoplasm only emerged from CD62L-neg

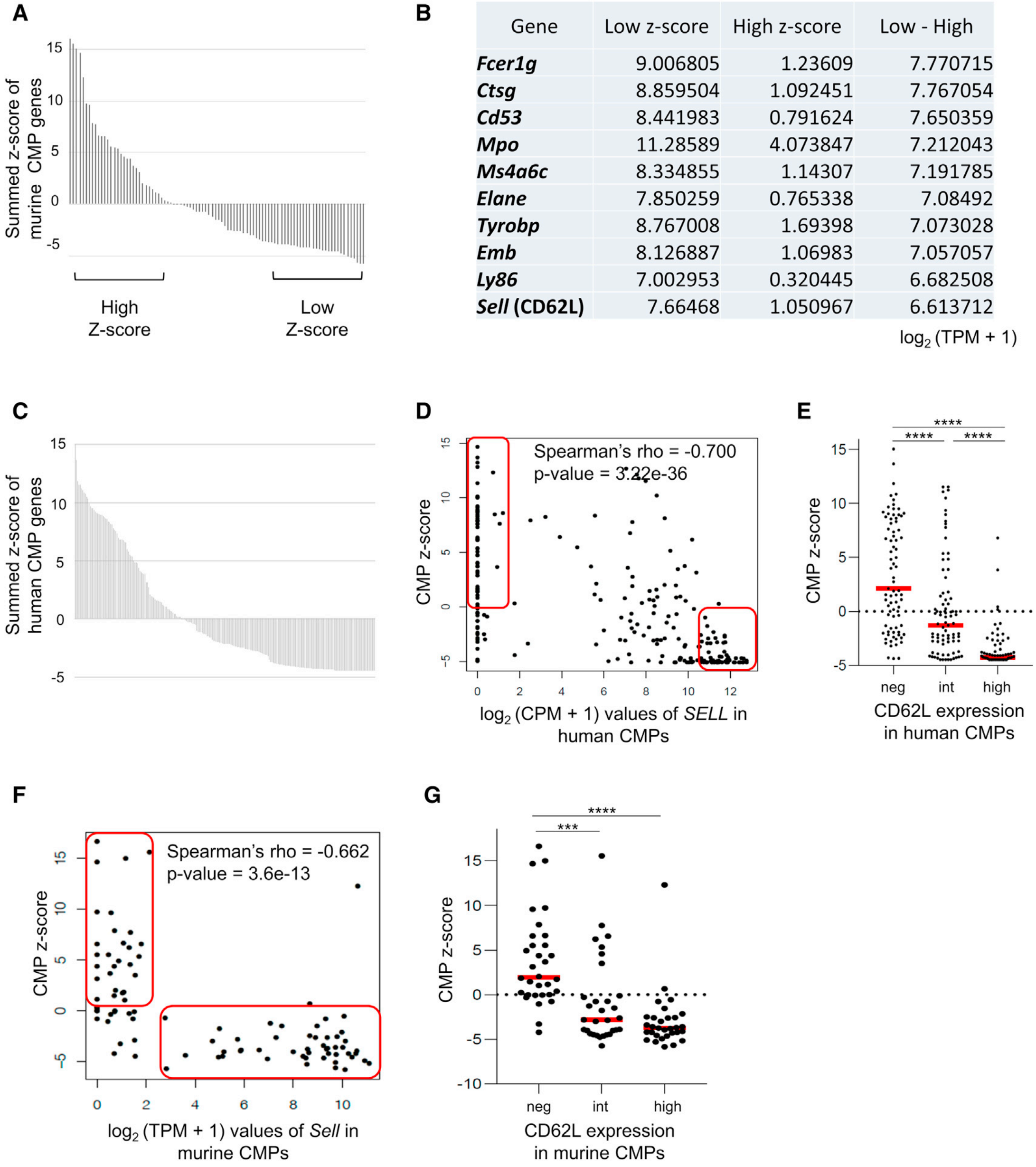

Gene	Low z-score	High z-score	Low - High
Fcer1g	9.006805	1.23609	7.770715
Ctsg	8.859504	1.092451	7.767054
Cd53	8.441983	0.791624	7.650359
Mpo	11.28589	4.073847	7.212043
Ms4a6c	8.334855	1.14307	7.191785
Elane	7.850259	0.765338	7.08492
Tyrobp	8.767008	1.69398	7.073028
Emb	8.126887	1.06983	7.057057
Ly86	7.002953	0.320445	6.682508
***Sell* (CD62L)**	7.66468	1.050967	6.613712

$\log_2$ (TPM + 1)

Figure 1. CD62L expression reveals heterogeneity of the CMP population

(A) The distribution of the sum of Z scores of ten CMP signature genes in individual murine CMP cells calculated from the publicly available single-cell RNA-seq data (GSE70236). The y axis shows the summed Z scores. Cells within the top and bottom third of the scores were defined as CMP-high and CMP-low groups, respectively.

(B) The list of the ten genes whose expressions is most upregulated in the CMP-low group compared with the CMP-high group.

(C) The distribution of the sum of Z scores of the ten CMP signature genes in each human CMP from the data of single-cell RNA-seq (GSE113046). The y axis shows the value of the sum of CMP *Z* score.

(D and F) Expression levels of *SELL*, the gene encoding CD62L, were plotted against the sum of CMP Z scores in individual cells in humans (D) and mice (F). The x axis shows the expression value of *SELL* and the y axis shows the sum of Z scores.

(E and G) The plot of the *Z* score divided by CD62L-neg, CD62L-int, and high CMPs in humans (E) and mice (G). Statistical significance was calculated using one-way ANOVA (***p < 0.001, ****p < 0.0001).

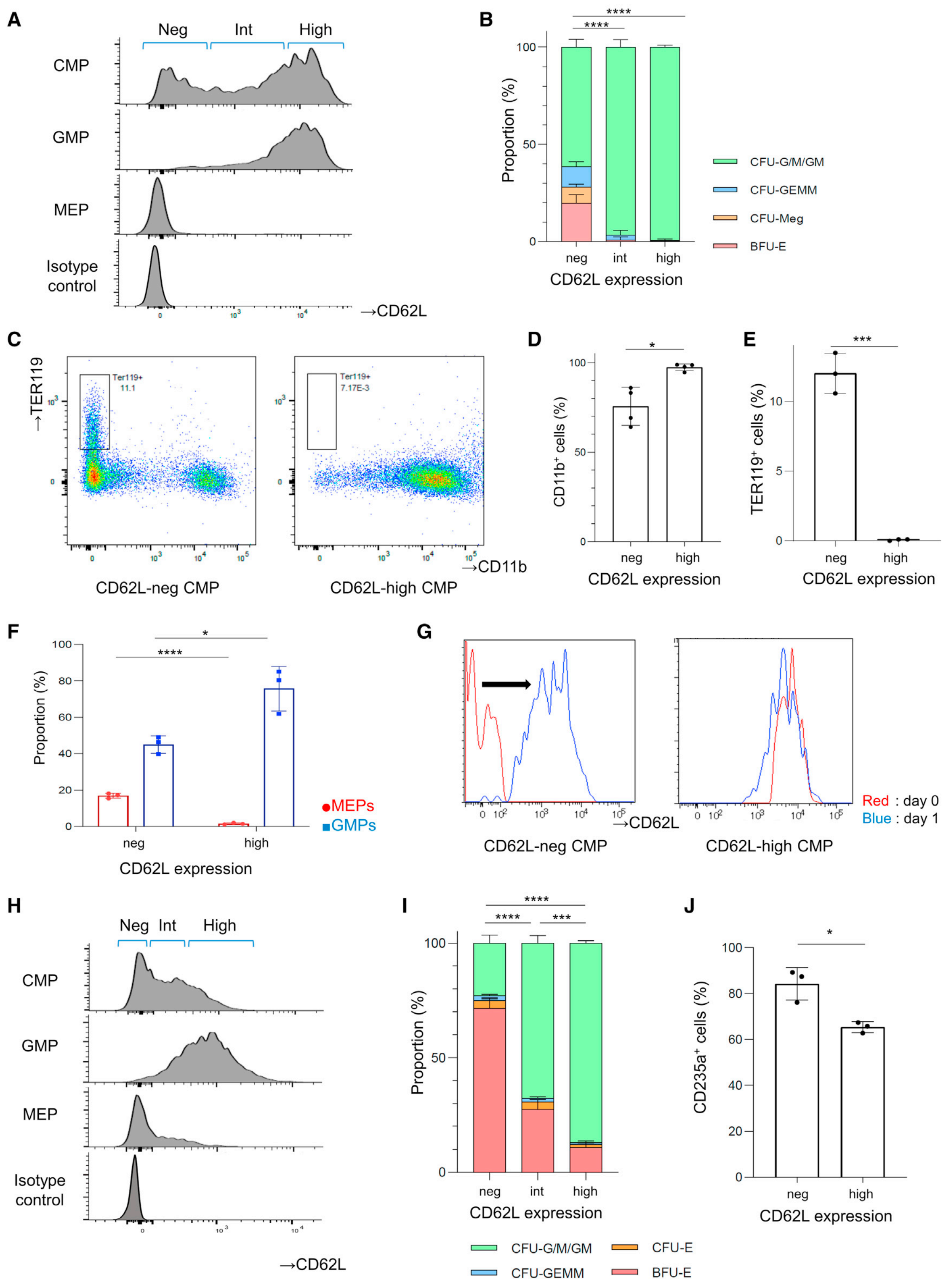

Figure 2. CD62L expression reveals the heterogeneity of CMPs *in vitro* both in mice and humans

(A) Representative flow cytometry plots analyzing CD62L expression or isotype control in each progenitor population (CMP, GMP, and MEP) within mouse bone marrow cells. CMPs were trisected according to CD62L expression level and defined as CD62L-neg, CD62L-int, and high CMPs.

(legend continued on next page)

CMPs (Figure 3A). We then investigated *in vivo* differentiation potential of the CD62L-high and CD62L-neg CMPs using mouse bone marrow transplantation assays (Okabe et al., 1997). Wild-type C57BL/6 mice were transplanted with CD62L-neg, CD62L-int, or high CMPs derived from the CAG-EGFP mice, and chimerism of the donor cells in the peripheral blood, bone marrow, and spleen was analyzed on day 7 (Figure 3B). Platelets were detected as FSC-low and CD41-positive fractions, and the frequency as well as the absolute count of GFP-positive platelets was significantly higher in mice transplanted with CD62L-neg CMPs (CD62L-neg CMPs, 9.8% ± 2.7%; CD62L-int CMPs, 0.4% ± 0.2%; CD62L-high CMPs, 0.2% ± 0.1%) (Figures 3C–3F). With regard to the localization of engrafted GFP-positive megakaryocytes, CD11b^{-}CD41^{+} cells were detected in splenic cells (Figure S3A). The proportion of GFP-positive neutrophils and macrophages was not significantly different among CD62L-neg, CD62L-int, and high CMP cells in peripheral blood, bone marrow, and spleen (Figures 3G–3K, S3B–S3E, and S3G–S3I). The proportion of TER119^{+} erythrocytes and platelets in the spleen was significantly higher in mice transplanted with CD62L-neg CMPs (Figures S3F, S3J, and S3K). These *in vivo* results reinforce that CD62L-high CMPs have lost differentiation potential into the megakaryocytic lineage.

Gene expression analysis for CD62L-neg and CD62L-high CMPs

To further corroborate the differentiation potential from the aspect of gene expression, we performed RNA-seq of murine CD62L-neg and CD62L-high CMPs, bulk CMPs and bulk GMPs. Principal-component analysis (PCA) showed a clear separation of CD62L-neg CMPs and CD62L-high CMPs (Figure 4A). Unsupervised hierarchical clustering showed that CD62L-high CMPs clustered adjacent to GMPs and away from the CD62L-neg CMPs (Figure 4B). Overall, 927 genes were upregulated and 1,064 genes were downregulated in CD62L-high CMPs significantly compared with CD62L-neg CMPs (fold change >2, FDR < 0.05, Figure S4A). Gene ontology analysis showed that differentially expressed genes in CD62L-high CMPs were enriched with immune system process, immune response, and cytokine production, whereas CD62L-neg CMPs highly expressed the cell adhesion pathway (Figure 4C). We performed k-means clustering of these four populations and divided genes into three clusters (Figure S4B). Pathway enrichment analysis revealed that genes within cluster A, which are enriched in CD62L-high CMPs and bulk GMPs, were related with immune reaction against infection, such as immune system process, immune response, and cytokine production, while pathways associated with cell adhesion and platelet function, such as blood coagulation, were upregulated in cluster B, which were enriched in CD62L-neg and bulk CMP (Figure S4C). These results are compatible with our functional assays; CD62L-high CMPs have acquired gene expression profiles representing monocytes and neutrophils while loss of expression of genes upregulated in megakaryocytes.

We also focused on expression levels of individual genes characteristic of each lineage. *Gata1*, *Zfpm1* (*Fog-1*), *Tal1* (*Scl*), *Lmo2*, *Klf1* (*Eklf*) (Cantor and Orkin, 2002; Perry and Soreq, 2002), and *Gfi1b* (Osawa et al., 2002) are essential transcription factors to erythroid and megakaryocytic differentiation and expansion. Erythropoietin receptor (*Epor*) and transferrin receptor 2 (*Tfr2*) (Forejtnikovà et al., 2010), *Gypa* (CD235a), *Tspo2* (Kiatpakdee et al., 2020), *Ldb1*, and carbonic anhydrase (*Car1*, *Car2*) (Song et al., 2012) are genes specifically expressed in erythroid-lineage cells. *Itga2b* (CD41), *Gp9* (CD42a), *Gp1ba* (CD42b), *Gp1bb* (CD42c), *Gp5* (CD42d), *Itgb3* (CD61) (Drexler et al., 1997), *Mpl*, *vWf*, and *Pf4* (Olsson et al., 2016) are representative megakaryocytic genes. These

(B) The result of colony-forming cell assay of murine CD62L-neg, CD62L-int, and high CMPs. Means ± SD of three independent experiments. Statistical significance for the proportion of CFU-G/M/GM was calculated using one-way ANOVA (****$p < 0.0001$; n = 3).
(C) Representative flow cytometry plots analyzing CD11b and TER119 expression after 7-day culture of CD62L-neg and high CMPs with semisolid medium.
(D and E) The result of differentiation into CD11b-positive cells (D) and TER119-positive cells (E) after 7-day culture. Means ± SD of three to four independent experiments. Statistical significance was calculated using unpaired two-tailed t test (*$p < 0.05$, ***$p < 0.001$; n = 3–4).
(F) The result of differentiation into GMPs and MEPs after 2-day liquid culture of CD62L-neg and CD62L-high CMPs. Means ± SD of three independent experiments. Statistical significance was calculated using unpaired two-tailed t test (*$p < 0.05$, ****$p < 0.0001$; n = 3).
(G) Representative flow cytometry plots analyzing CD62L expression when CD62L-neg and CD62L-high CMPs were sorted (red) and after 1-day liquid culture (blue). Experiments were repeated three times.
(H) Representative flow cytometry plots analyzing CD62L expression or isotype control in each progenitor population (CMP, GMP, and MEP) within human CD34^{+} bone marrow cells.
(I) The result of colony-forming cell assay of human CD62L-neg, CD62L-int, and high CMPs. Means ± SD of three independent experiments. Statistical significance for the proportion of CFU-G/M/GM was calculated using one-way ANOVA (***$p < 0.001$, ****$p < 0.0001$; n = 3).
(J) The result of proportion of CD235a-positive erythroid cells after 14-day culture with semisolid medium. Means ± SD of three independent experiments. Statistical significance was calculated using unpaired two-tailed t test (*$p < 0.05$; n = 3).

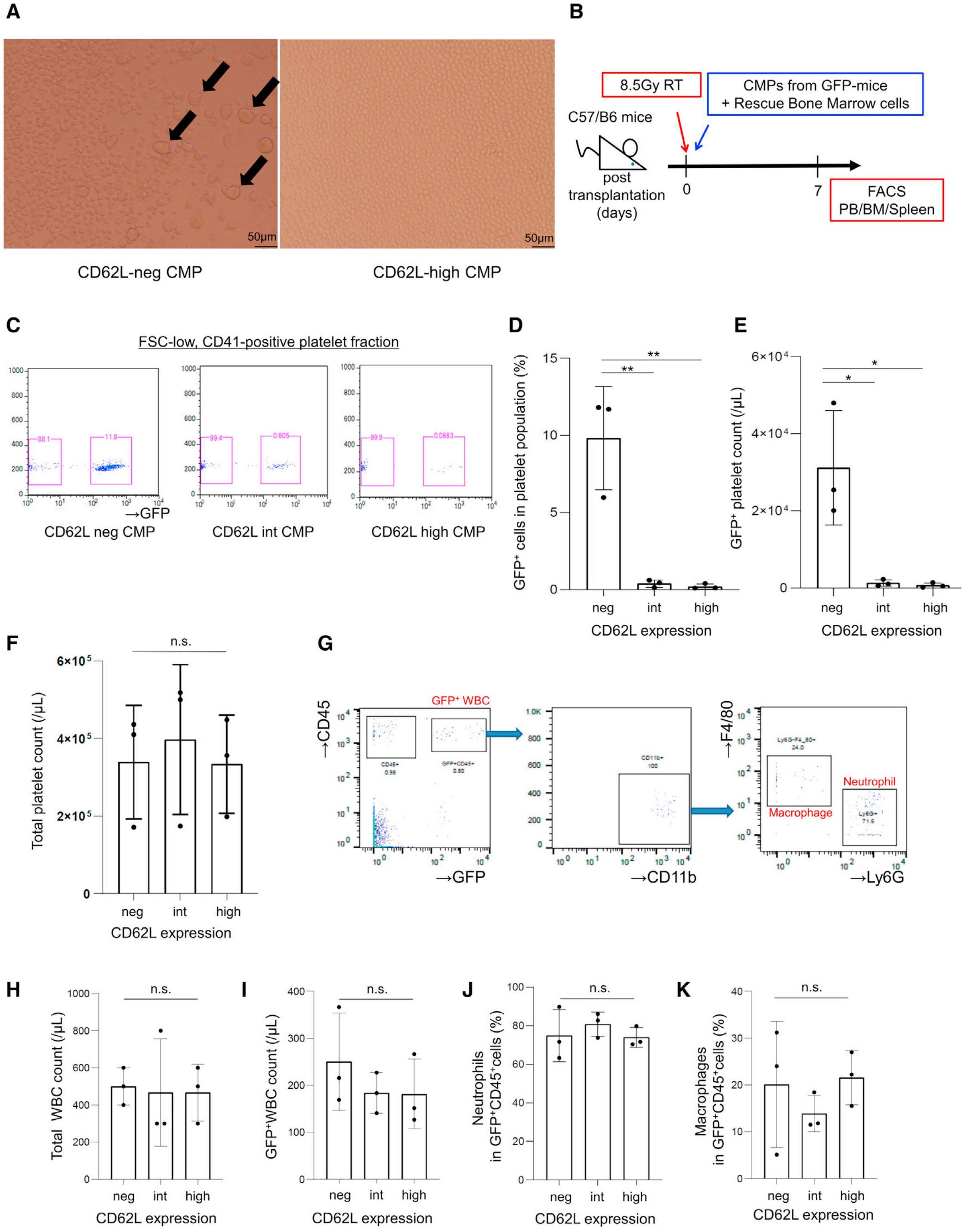

Figure 3. CD62L-neg but not CD62L-high CMPs differentiate into megakaryocytic cells *in vitro* and *in vivo*

(A) The microscopic findings after 4-day liquid culture of CD62L-neg and CD62L-high CMPs. Large megakaryocytes were exclusively differentiated from CD62L-neg CMPs (arrows).

(B) A scheme of *in vivo* transplantation assay. Wild-type mice were lethally irradiated (8.5 Gy), and then 1.5×10^4 CD62L-neg, CD62L-int, or high CMPs from CAG-EGFP mice and 2×10^5 bone marrow cells from wild-type mice were injected intravenously. Peripheral blood, bone marrow, and spleen were analyzed after 7 days.

(legend continued on next page)

genes were all significantly upregulated in CD62L-neg CMPs compared with CD62L-high CMPs (Figure 4D). On the other hand, *Spi1*, *Cebpa*, *Cebpb*, *Cebpe*, *Gfi1* (Olsson et al., 2016), *Irf8*, and *Klf4* (Kurotaki et al., 2013) are essential transcription factors for differentiating into neutrophils and monocytes. M-CSFR (*Csf1r*, CD115), GM-CSFR (*Csf2rb*, CD131), and G-CSFR (*Csf3r*, CD114) are important receptors for granulocytic and monocytic colony-stimulating factors. *Ctsg*, *Mpo*, *Elane*, *Slpi*, and *Ly86* are specific genes for granulocytes and monocytes (Olsson et al., 2016). Expression of these genes was significantly higher in CD62L-high CMPs than CD62L-neg CMPs (Figure 4E). These profiles were confirmed at single-cell level by using published murine and human single-cell RNA-seq data (GSE70236 and GSE113046) (Drissen et al., 2019; Olsson et al., 2016) (Figures S4D–S4I and S5A–S5H). Moreover, we compared our RNA-seq data of bulk CMPs and bulk GMPs, and created a top 200 CMP signature gene sets and top 200 GMP signature gene sets (Table S1). Gene set enrichment analysis (GSEA) showed that CD62L-neg CMPs had CMP signatures (Figure 4F) and that CD62L-high CMPs had GMP signatures (Figure 4G). Collectively, our data demonstrated that CD62L-neg CMPs have genuine CMP attributes and CD62L-high CMPs are skewed to the GMP population (Figure 4H).

CD62L-neg GMPs in mice are the most immature subset in GMPs

Next, we investigated whether a small population of CD62L-neg cells in the GMP fraction (Figure 2A) is functionally different from the CD62L-positive GMP in mice. Approximately 10% of the murine GMP cells were negative or dim positive for CD62L, thus we defined this lowest 10% as CD62L-neg GMPs and analyzed these subsets (Figure 5A). Colony-forming cell assay revealed that colonies derived from CD62L-neg GMPs were still CD11b negative after 7 days of culture (Figures 5B and 5C), and that these CD11b-negative cells were almost TER119 negative, suggesting immature myeloid cells.

To follow the transition of CD62L expression on GMPs, we analyzed the expression of CD62L in 24 h after liquid culture, which showed that CD62L-neg GMPs were only generated from CD62L-neg CMPs and that CD62L-neg GMPs differentiated into CD62L-positive GMPs but not vice versa (Figure 5D). These results suggest that CD62L-neg GMPs are the most immature subset in GMPs, and that CD62L-neg CMPs differentiate into CD62L-pos GMPs through two pathways: CD62L-high CMPs or CD62L-neg GMPs, depending on the order of upregulation of CD62L and CD16/32. To confirm these results, we analyzed single-cell RNA-seq data of murine GMPs (GSE130966 and GSE70240) (Dress et al., 2019; Olsson et al., 2016). Consistent with our *in vitro* data, calculating CMP Z scores using both data demonstrated that only a few GMPs had high CMP signatures and none of them expressed CD62L (Figures 5E and 5F). These data suggest that CD62L-neg GMPs are the most immature subsets in GMPs, located between CMPs and GMPs in mice.

To further elucidate the characteristics of CD62L-neg GMPs, we performed RNA-seq of murine progenitors. PCA and hierarchical clustering showed that CD62L-neg GMPs formed a distinct subset in GMPs (Figures 6A and 6B). Overall, 483 genes were significantly upregulated and 418 genes were downregulated in CD62L-neg GMPs compared with bulk GMPs (Figure 6C). Heatmaps showed that some essential genes to erythroid and megakaryocytic differentiation were still expressed in CD62L-neg GMPs (Figure 6D), while some genes to granulocytic and monocytic differentiation were not yet fully upregulated (Figure 6E). GSEA showed that CMP signature genes were downregulated in order of bulk CMPs, CD62L-neg GMPs, and bulk GMPs (Figures 6F and 6G), whereas GMP signature genes were upregulated in order of bulk CMPs, CD62L-neg GMPs, and bulk GMPs (Figures 6H and 6I). These results were further supported at the single-cell level (GSE130966), which demonstrated that some CD62L-neg GMPs highly expressed *Gata1*, *Klf1*, *Gfi1b* (which are expressed higher in conventional CMPs) and some did not express *Spi1*, *Irf8*, *Cebpa*, *Cebpb*, and *Runx1* (which are expressed higher in conventional GMPs) (Figures S5I–S5P). Collectively, these RNA-seq data clarified that CD62L-neg GMPs were the most immature subsets in GMPs and located between CMPs and GMPs, and still possessed gene expression patterns of CMPs to some extent in mice.

(C) Representative flow cytometry plots analyzing GFP expression in FSC-low, CD41-positive platelets from each mouse. Experiments were repeated three times.

(D–F) The result of proportion (D) and absolute count (E) of GFP-positive platelets, and (F) total platelet count in peripheral blood from each mouse. Means ± SD of three independent experiments. Statistical significance was calculated using one-way ANOVA (*$p < 0.05$, **$p < 0.01$; n.s., not significant, n = 3).

(G) Representative flow cytometry plots analyzing Ly6G^{+} neutrophils and F4/80^{+} macrophages in GFP-positive cells from each mouse. Experiments were repeated three times.

(H–K) The result of (H) total CD45^{+} cell count, (I) GFP-positive cell count, and the proportion of (J) neutrophils and (K) macrophages in GFP-positive cells in peripheral blood. Means ± SD of three independent experiments. Statistical significance was calculated using one-way ANOVA (n.s., not significant, n = 3).

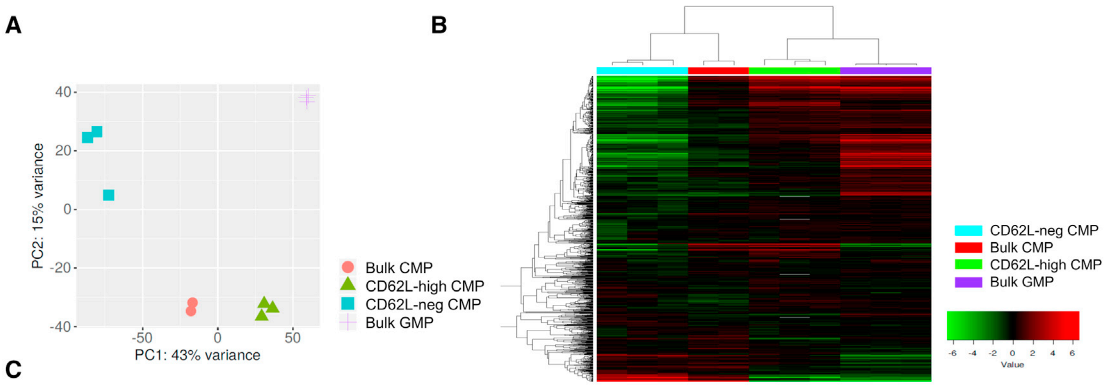

C

	p-value	Enriched GO terms in DEGs		p-value	Enriched GO terms in DEGs
**Enriched in CD62L-high CMPs**	1.3e-47	Immune system process	**Enriched in CD62L-neg CMPs**	3.0e-19	Cell adhesion
	5.5e-42	Immune response		3.0e-19	Biological adhesion
	5.6e-35	Defense response		1.2e-18	Regulation of biological quality
	9.8e-31	Regulation of immune system process		9.5e-17	Negative regulation of cellular process
	1.0e-30	Response to external stimulus		1.6e-16	Regulation of response to stimulus
	1.2e-28	Positive regulation of immune system process		9.3e-16	Regulation of signaling
	1.3e-28	Cytokine production		1.1e-15	Regulation of localization
	1.7e-28	Response to stress		1.1e-15	Regulation of multicellular organismal process
	2.9e-26	Regulation of response to stimulus		1.1e-15	Regulation of cell communication
	1.3e-25	Regulation of immune response		2.3e-15	System development

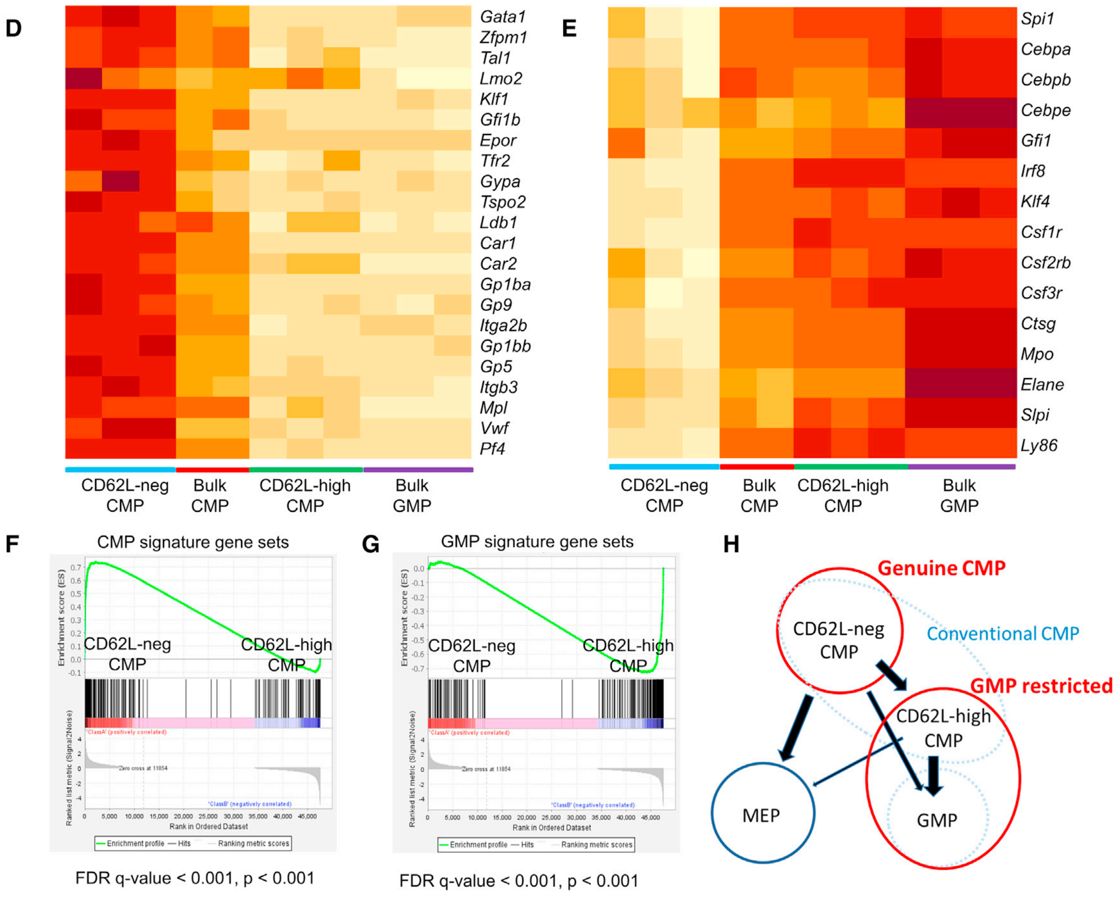

(legend on next page)

CD62L-int GMPs are skewed to neutrophil differentiation in mice

Although GMPs are defined as cells that can differentiate into both neutrophils and monocytes, several studies showed the existence of subsets destined to differentiate into either neutrophils or monocytes alone (Kwok et al., 2020; Yáñez et al., 2015). We tested whether the difference in CD62L expression levels within the GMP population is associated with their heterogeneous differentiation potential in mice. The majority of murine GMP cells ranged from intermediate to high expression levels, and we isolated the highest 10% (high levels) and the lowest 10% (intermediate levels) (Figure 7A). Colony-forming cell assay showed that CD62L-int GMPs were skewed to neutrophil differentiation (CFU-G: 74.7% ± 6.2%, CFU-M: 17.5% ± 3.4%) compared with CD62L-high GMPs (CFU-G: 44.9% ± 2.8%, CFU-M: 45.6% ± 3.9%) (Figure 7B). F4/80-positive macrophages (Figure S6A) were more abundant in CD62L-high GMPs (10.1% ± 1.3% versus 5.3% ± 0.4%, Figures 7C and 7D). To confirm these findings *in vivo*, we performed transplantation assay using Ly5.1 mice as donors and Ly5.2 mice as recipients. We transplanted CD62L-int, CD62L-high, or bulk GMPs from Ly5.1 mice into lethally irradiated Ly5.2 mice (Figure 7E). On day 5, the analysis of splenic cells revealed that CD62L-int GMPs differentiated into Ly6G-positive neutrophils significantly more than the bulk and CD62L-high GMPs (Figures 7F, 7G, and S6C), while donor-derived cells were scarcely detected in the peripheral blood and bone marrow (Figures S6B and S6D–S6G).

We then performed RNA-seq of bulk, CD62L-int, and CD62L-high GMPs. PCA and clustering analysis showed their heterogeneity (Figures 7H, 7I, and S6H). We focused on some neutrophil- or monocyte-specific genes. *Gfi1* and *Cebpe* are important transcription factors for neutrophil fate choice, while *Irf8* and *Klf4* for monocyte (Kurotaki et al., 2013; Laurenti and Göttgens, 2018). Also, *Vcam1*, *Rgcc*, *Gfi1*, and *Cd81* were known as neutrophil-related genes, and *Csf1r*, *Ly86*, and *Irf8* as monocytic fate-determining genes (Kwok et al., 2020). We analyzed these expressions in our RNA-seq data and confirmed the tendency that neutrophil-related genes were upregulated in CD62L-int GMPs, whereas monocyte-related genes were downregulated (Figure 7J).

Previous reports showed that Ly6C-neg GMPs were more immature oligopotent progenitors in GMPs than Ly6C-positive GMPs (Yáñez et al., 2015). When Ly6C and CD62L expressions in GMPs were analyzed, CD62L-neg GMPs were all included in Ly6C-neg GMPs, which was compatible that CD62L-neg GMPs were the most immature subsets in GMPs (Figure 7K). Also, Ly6C-positive GMPs were able to be divided into CD62L-int and CD62L-high GMPs, and $Ly6C^+$CD62L-int GMPs were more skewed to neutrophil differentiation, while $Ly6C^+$CD62L-high GMPs were significantly skewed to monocyte differentiation ($Ly6C^+$CD62L-int GMPs: CFU-G 81.1% ± 5.3%, CFU-M 15.6% ± 4.8%, $Ly6C^+$CD62L-high GMPs: CFU-G 34.4% ± 6.3%, CFU-M 56.2% ± 4.1%, Figure 7L). Consequently, these experiments revealed that CD62L can be used as a robust surface marker for subdividing heterogeneous murine myeloid progenitors (Figure S7).

DISCUSSION

Recent studies have revealed the heterogeneity in each myeloid progenitor. For instance, in humans, the CD64-high/CLEC12a-high fraction in GMPs was identified as monocyte-restricted progenitors (Kawamura et al., 2017), and $CD41^+$ CMPs as unipotent megakaryocyte progenitors (Miyawaki et al., 2017). On the other hand, in mice, $Ly6C^+CD115^+$ GMPs were determined as monocyte progenitors (Yáñez et al., 2015), and $CD42b^+$ CMPs as unipotent megakaryocyte progenitors (Nishikii et al., 2015). Here, we studied the heterogeneity of CMPs by using single-cell RNA-seq data. We demonstrated that previously defined CMPs contained the subset, which was skewed to GMP differentiation potential, and CD62L was a useful marker to distinguish this subgroup. These findings suggest that significant heterogeneity has been offset by putting these subsets together in the previous data of bulk CMPs. To separate subsets and purify more homogeneous and

Figure 4. Gene expression profiles of murine CD62L-neg and CD62L-high CMPs

(A) Principal-component analysis of bulk CMPs, CD62L-neg CMPs, CD62L-high CMPs, and bulk GMPs.

(B) The result of hierarchical clustering of bulk CMPs, CD62L-neg CMPs, CD62L-high CMPs, and bulk GMPs using the top 1,000 genes ranked by their standard deviation.

(C) The result of gene ontology analysis of differentially expressed genes between CD62L-neg CMPs and CD62L-high CMPs. The top 10 enriched gene ontology terms are listed.

(D and E) Heatmap of representative genes essential for erythrocytes and megakaryocytes (D) and granulocytes and monocytes (E).

(F and G) Gene set enrichment analysis of the CMP signature gene set (F) and the GMP signature gene set (G) comparing the indicated populations.

(H) Proposed model of CMP differentiation. CD62L-neg CMPs are located at the upper differentiation hierarchy, and CD62L-high CMPs are more differentiated into GMP population.

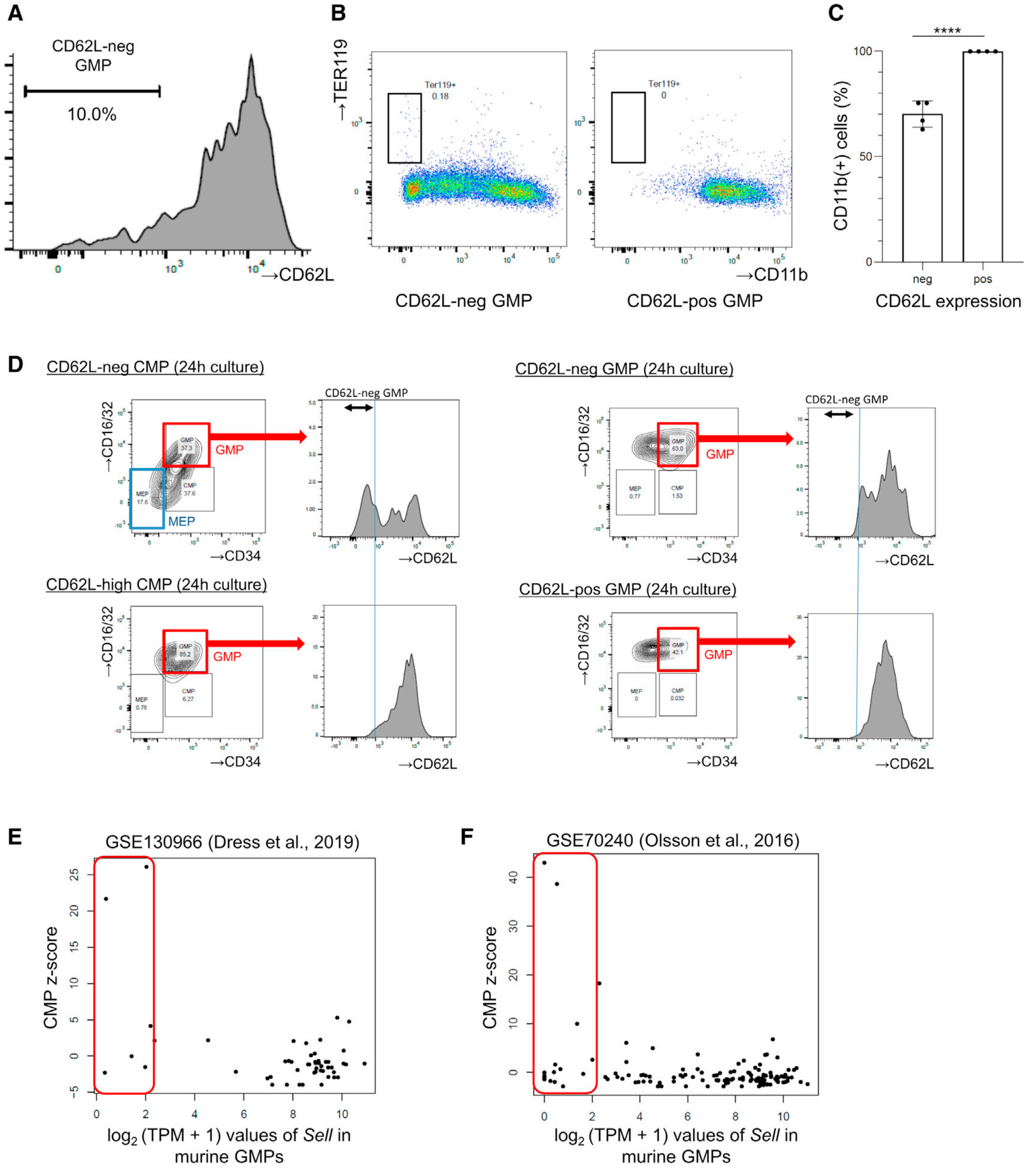

Figure 5. The CD62L-neg cell population is the most immature subset within murine GMPs

(A) Representative flow cytometry plots analyzing CD62L expression in murine GMPs. CD62L-neg GMPs are defined as low 10% population.

(B) Representative flow cytometry plots analyzing CD11b and TER119 expression after 7-day culture with semisolid medium. Experiments were performed four times.

(C) The result of differentiation into CD11b-positive cells after 7-day culture. Means ± SD of four independent experiments. Statistical significance was calculated using unpaired two-tailed t test (****$p < 0.0001$; $n = 4$).

(D) Representative flow cytometry plots analyzing CD62L expression in GMPs after 1-day culture of CD62L-neg CMPs, CD62L-high CMPs, CD62L-neg GMPs, and CD62L-pos GMPs. Experiments were performed three times.

(E and F) The relationship between the summed *Z* score of CMP genes and CD62L expression from single-cell RNA-seq data of murine GMPs (E) (GSE130966) and (F) (GSE70240). The x axis shows $\log_2$(TPM + 1) of CD62L and the y axis shows the summed *Z* score of CMP signature genes.

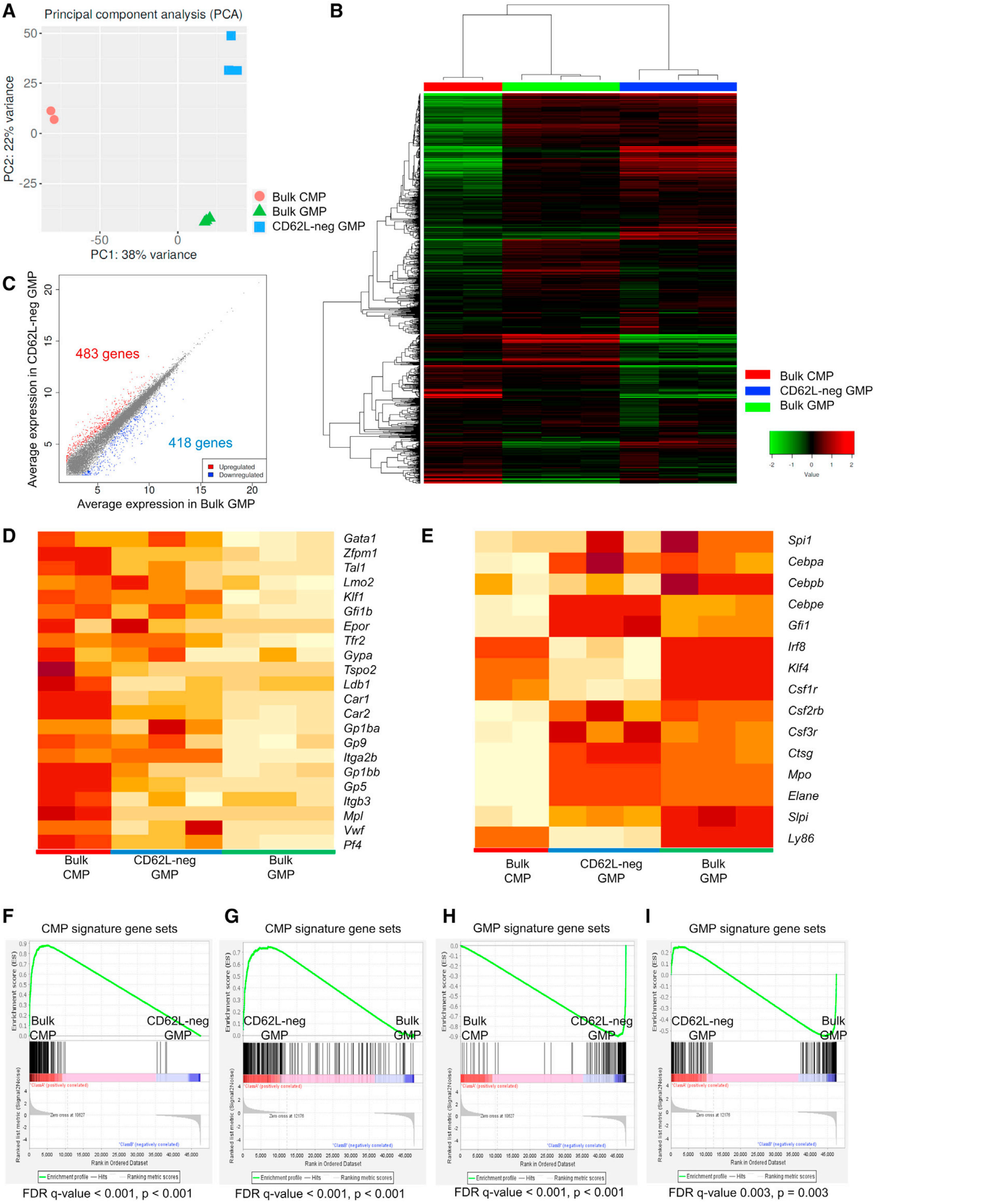

Figure 6. Gene expression profiles of CD62L-neg GMPs

(A) Principal-component analysis of bulk CMPs, CD62L-neg GMPs, and bulk GMPs.

(B) The result of hierarchical clustering of bulk CMPs, CD62L-neg GMPs, and bulk GMPs.

(C) The scatterplot of differentially expressed genes between CD62L-neg GMPs and bulk GMPs. Fold change > 2, FDR < 0.05.

(D and E) Heatmap of representative genes essential for erythrocytes and megakaryocytes (D), and granulocytes and monocytes (E).

(F–I) Gene set enrichment analysis of CMP signature (F and G) and GMP signature (H and I) gene sets comparing the indicated populations.

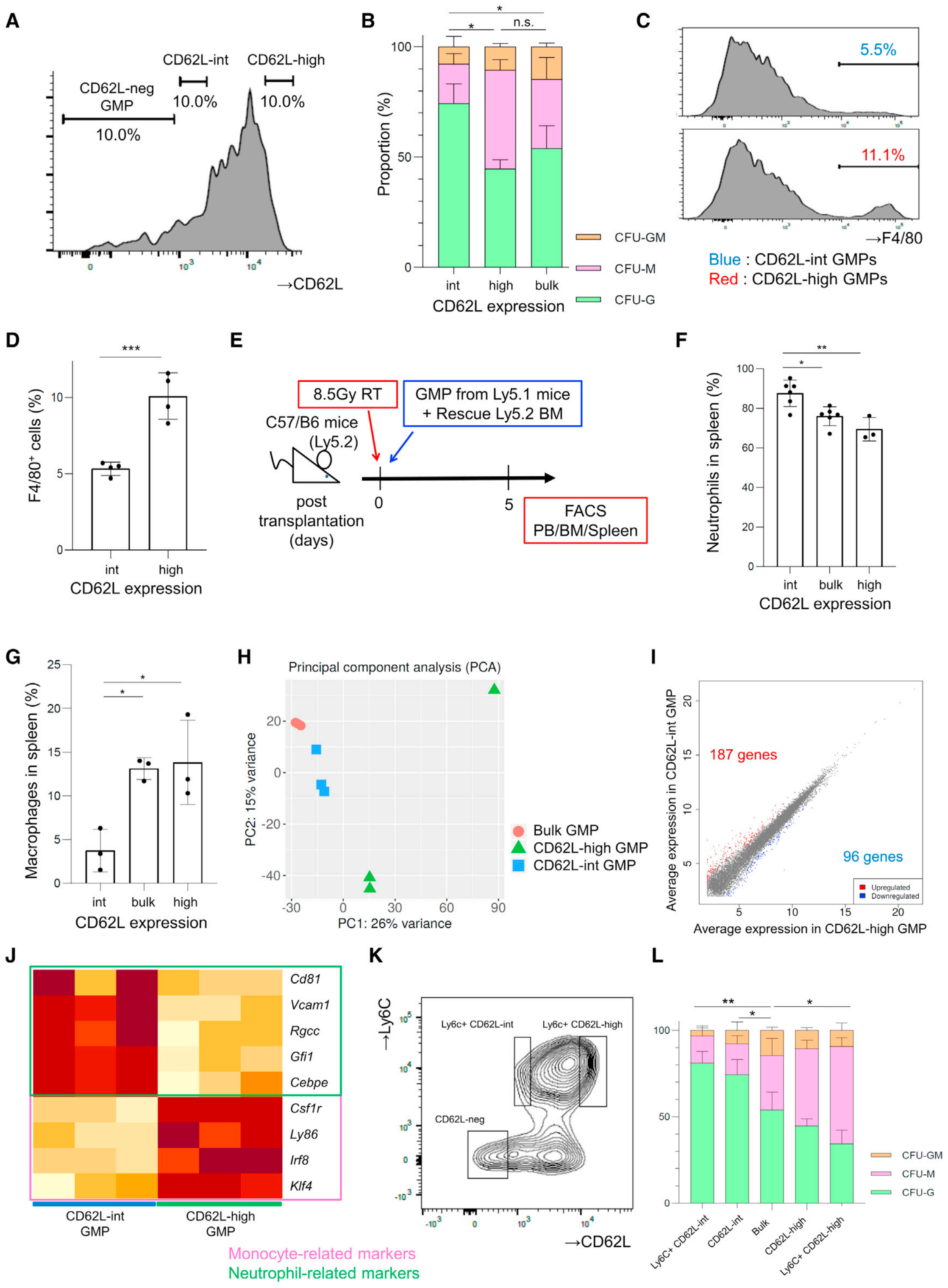

Figure 7. GMPs possess different differentiation potential into neutrophils and monocytes according to CD62L expression levels

(A) Representative flow cytometry plots analyzing CD62L expression in GMPs within mouse bone marrow cells with the sorting strategy of CD62L-int and CD62L-high cells.

(legend continued on next page)

genuine CMPs enable to determine their characteristics more precisely, and elucidate more sophisticated mechanism of myeloid differentiation. Moreover, CD62L expression in CMPs shows the same tendency in mice and humans, which makes it easier to apply the results of mice to humans.

We elucidated the heterogeneity of GMPs and found that CD62L-neg GMPs were the most immature subgroups in GMPs. Our results suggest that a small part of them may still possess CMP potential, which may suggest the incompleteness of the present surface markers to define myeloid differentiation potential and should be further elucidated. Also, we showed the existence of neutrophil- and monocyte-skewed populations in GMPs, which was compatible with several former studies (Dinh et al., 2020; Kwok et al., 2020), and added new aspects for myeloid differentiation. We confirmed that CD62L-high CMPs differentiate into GMPs while maintaining the positive expression of CD62L, which suggests that CD62L-positive GMPs can be generated from both CD62L-neg GMPs and CD62L-high CMPs. Further investigation is needed to clarify the difference between these differentiation pathways.

CD62L is an adhesion molecule, also called as L-selectin (SELL), and its function has been mainly studied in T cells. CD62L on T cells is important for T cell homing and associated with T cell quiescence (Ivetic et al., 2019). Until now, the role of CD62L on hematopoietic progenitors was only studied in multipotent progenitors (MPPs) (Cho and Spangrude, 2011) and T cell-lineage progenitors (Perry et al., 2004). These studies revealed the heterogeneity of Lin^{-}SCA-1^{+}C-KIT^{+} (LSK) population by CD62L. In LSK fraction, CD62L-low subsets were more immature and mainly contained long-term hematopoietic stem cells, whereas CD62L-high subsets contained MPPs, and also upregulation of CD62L in MPPs decreased erythro-megakaryocytic lineage potentials (Cho and Spangrude, 2011), which were compatible with our data. However, CD62L expression on myeloid progenitors has not been fully examined so far. CD62L is also an important adhesion molecule in neutrophils and highly expressed on mature neutrophils (Tak et al., 2017). Monocytes also express CD62L, but the expression level in mice is lower than neutrophils (data not shown). Therefore, it is needed to elucidate the expression transition and regulation mechanism between unipotent progenitors and mature cells.

In conclusion, we identified CD62L as a surface marker to elucidate the heterogeneity of CMPs and GMPs, and refined these differentiation potentials. CD62L-neg CMPs are genuine CMPs, whereas CD62L-high CMPs are highly restricted to GMP potentials in mice and humans. CD62L-neg GMPs are the most immature subset in GMPs, and Ly6C^{+}CD62L-int and CD62L-high GMPs are skewed to neutrophil and monocyte differentiation, respectively in mice. These findings refine the definition of CMPs and GMPs, and elucidate the differentiation mechanism of myeloid cells in more detail.

EXPERIMENTAL PROCEDURES

Mice

C57BL/6 mice (Ly5.2), C57BL/6-CD45.1 (Ly5.1), and C57BL/6-Tg(CAG-EGFP)C14-Y01-FM131Osb mice were purchased from Japan SLC, Sankyo Lab Service Corporation, and RIKEN Bio-Resource Research Center, respectively (Okabe et al., 1997). All mice were aged 8–12 weeks when used for experiments. All animal experiments were approved by The University of Tokyo Ethics Committee for Animal Experiments and performed according to the Guidelines for Animal Experiments of the University of Tokyo.

(B) Colony-forming cell assay of murine CD62L-int, CD62L-high, and bulk GMPs. Mean proportions ± SD of three independent experiments for the indicated colonies are shown. Statistical significance for the proportion of CFU-G was calculated using one-way ANOVA (*$p < 0.05$; n.s., not significant; n = 3).
(C and D) F4/80 expression was analyzed after 7-days culture of the indicated populations in semisolid medium. Representative flow cytometry plots (C) and frequency of the F4/80^{+} cells are shown (D) (n = 4; unpaired t test; ***$p < 0.001$).
(E) A scheme of *in vivo* transplantation assay. Lethally irradiated (8.5 Gy) Ly5.2 mice were intravenously injected with 1.0–4.0 × 10^{4} CD62L-int, CD62L-high, or bulk GMPs derived from Ly5.1 mice with 2.0 × 10^{5} bone marrow cells from Ly5.2 mice. Differentiation of the transplanted cells was analyzed 5 days after transplantation.
(F and G) Differentiation of transplanted GMPs into neutrophils and macrophages in splenic cells was analyzed by flow cytometry. The frequency of (F) neutrophils and (G) macrophages in the Ly5.1-positive donor cell population is shown. Means ± SD of three to six independent experiments. Statistical significance was calculated using unpaired one-way ANOVA (*$p < 0.05$, **$p < 0.01$).
(H) Principal-component analysis of gene expression profiles among CD62L-int, CD62L-high, and bulk GMPs.
(I) The scatterplot of differentially expressed genes between CD62L-int GMPs and CD62L-high GMPs. Fold change > 2, FDR < 0.05.
(J) Heatmap of representative genes important for granulocyte and monocyte differentiation.
(K) Representative flow cytometry plot analyzing CD62L and Ly6C expression in murine GMPs. Experiments were performed three times.
(L) The result of colony-forming cell assay of murine Ly6C^{+}/CD62L-int and Ly6C^{+}/CD62L-high GMPs. Mean proportions ± SD of three independent experiments. Statistical significance for the proportion of CFU-G was calculated using one-way ANOVA (*$p < 0.05$, **$p < 0.01$; n = 3).

Colony-forming cell assay

For colony-forming cell assay, murine myeloid progenitors were cultured at 1 × 10^3 cells per well for 7 days with 1 mL of methylcellulose medium (MethoCult GF M3434, STEMCELL Technologies). $CD34^+$ human bone marrow samples were purchased from Lonza, and myeloid progenitors were cultured at 1 × 10^3 cells per well for 14 days in methylcellulose medium (MethoCult H4434 Classic, STEMCELL Technologies).

Liquid culture assay

For liquid culture, murine myeloid progenitors were cultured at 1–2 × 10^3 cells per well in IMDM (Sigma) medium containing 20% FBS, 1% penicillin/streptomycin, and cytokines (50 ng/mL Flt3L, 50 ng/mL TPO, 100 ng/mL SCF, and 20 ng/mL IL-3) at 37°C in a 5% CO_2 incubator. The transition of CD62L expression level in CMPs and GMPs were analyzed after 24 h, and the differentiation capacity of CMPs into GMPs and MEPs were analyzed after 48 h.

In vivo transplantation assay

For *in vivo* transplantation assay of CMPs, C57BL/6 mice were irradiated at lethal doses (8.5 Gy) and intravenously infused with 1.5 × 10^4 CMP cells from CAG-EGFP mice and 2.0 × 10^5 bone marrow cells from C57BL/6 mice. One week after transplantation, peripheral blood, bone marrow, and spleen were analyzed. Blood cell count was performed by ERMA PCE-210N (ERMA). For *in vivo* transplantation of GMPs, C57/B6 mice were irradiated at lethal doses (8.5 Gy) and infused with 1.0–4.0 × 10^4 GMP cells from Ly5.1 mice and 2.0 × 10^5 bone marrow cells from C57BL/6 mice. Peripheral blood, bone marrow, and spleen were analyzed 5 days after transplantation.

Flow cytometry and cell sorting

Isolation of cells was performed using FACSAria II or III Cell Sorter (BD Biosciences). Data were analyzed with FlowJo (TreeStar, Ashland, OR, USA). To isolate murine progenitors, murine CMPs, GMPs, and MEPs were defined as $Lin^-SCA\text{-}1^-C\text{-}KIT^+CD16/32^-CD34^+$, $Lin^-SCA\text{-}1^-C\text{-}KIT^+CD16/32^+CD34^+$, and $Lin^-SCA\text{-}1^-C\text{-}KIT^+CD16/32^-CD34^-$ cells, respectively (Akashi et al., 2000). Biotinylated anti-GR-1 (RB6-8C5; BioLegend), CD11b (M1/70; BioLegend), TER119 (TER-119; BioLegend), B220 (RAS-6B2; BioLegend), CD3e (145-2C11; BioLegend), CD4 (GK1.5; BioLegend), CD8a (53-6.7; BioLegend), and CD127 (A7R34; BioLegend), followed by streptavidin-APC/Cy7 (BioLegend), PE/Cy7-conjugated anti-C-KIT (2B8; BioLegend), PerCP/Cy5.5 (D7; BioLegend), PE-conjugated anti-SCA-1 (D7; eBioscience), APC-conjugated anti-CD16/32 (93; BioLegend), FITC-conjugated anti-CD34 (RAM34; eBioscience), PE or Pacific blue-conjugated anti-CD62L (MEL-14; BioLegend), PerCP/Cy5.5-conjugated anti-Ly6C (HK1.4; BioLegend), and PE-conjugated rat IgG2aκ isotype control (BioLegend) were used. When CAG-EGFP mice were used, lineage marker-positive cells were depleted by streptavidin microbeads by AutoMACS Pro Separator (Miltenyi Biotec) at first, then PerCP/Cy5.5-conjugated anti-SCA-1 (D7; BioLegend), PE/Cy7-conjugated anti-C-KIT (2B8; BioLegend), APC/Cy7-conjugated anti-CD16/32 (93; BioLegend), Alexa Fluor 647-conjugated anti-CD34 (RAM34; BD Biosciences), and PE-conjugated anti-CD62L (MEL-14; BioLegend) were used. Human CMPs were purified as $Lin^-CD34^+CD38^+CD45RA^-CD123^{mid}$ populations (Manz et al., 2002). Biotinylated anti-CD3 (HIT3a; BioLegend), CD11b (ICRF44; BioLegend), CD14 (HCD14; BioLegend), CD16 (3G8; BioLegend), CD19 (HIB19; BioLegend), CD20 (2H7; BioLegend), CD56 (HCD56; BioLegend), and CD235a (HIR2; eBioscience), followed by streptavidin-PerCP/Cy5.5 (BioLegend), APC or PE/Cy7-conjugated anti-CD34 (581; BioLegend), APC/Cy7-conjugated anti-CD38 (HIT2; BioLegend), Pacific blue or APC-conjugated anti-CD123 (6H6; BioLegend), FITC-conjugated anti-CD45RA (HI100; BD Biosciences), PE-conjugated anti-CD62L (DREG-56; BioLegend), Pacific blue-conjugated anti-CD41 (HIP8; BioLegend), PE/Cy7-conjugated anti-CD71 (CY1G4; BioLegend), and PE-conjugated mouse IgG1κ isotype control (BioLegend) were used. For colony-forming cell assay, PE- or APC-conjugated anti-CD11b (M1/70; BioLegend), PE-conjugated anti-F4/80 (BM8.1; Tombo biosciences), APC-conjugated TER119 (TER-119, BioLegend), and PerCP/Cy5.5-conjugated anti-Ly6G (1A8; BioLegend) were used. For *in vivo* transplantation assay, APC-conjugated anti-CD41 (eBioMWReg30; eBioscience), APC-conjugated anti-CD45 (30-F11; BD Biosciences), PE/Cy7-conjugated CD45 (30-D11; BioLegend), FITC or PE/Cy7-conjugated anti-CD45.1 (A20; BioLegend), PE-conjugated anti-CD45.2 (104; BioLegend), PerCP/Cy5.5-conjugated anti-Ly6G (1A8; BioLegend), PE- or APC-conjugated CD11b (M1/70; BioLegend), PE- or PE/Cy7-conjugated F4/80 (BM8; BioLegend), and PE-conjugated TER119 (TER-119; BioLegend) were used.

RNA-seq

For gene expression profiling, RNA-seq was performed with the use of SMART-Seq v.4 Ultra Low Input. 2.5–4.0 × 10^4 cells of bulk CMPs, CD62L-neg CMPs, CD62L-high CMPs, bulk GMPs, CD62L-neg GMPs, CD62L-int GMPs, and CD62L-high GMPs were sorted per sample. All subsets were triplicated and one bulk CMP sample was excluded due to the low mapping rate. Base calling was performed using Illumina RTA software in sequencer, and further demultiplexing was performed using Illumina bcl2fastq software. The raw data were stored in FASTQ format. Trimming, mapping, and acquiring read count data were performed by Trim-galore, Hisat2, and Htseq. Differentially expressed gene analysis was performed using the DESeq2 package. PCA, hierarchical clustering, k-means clustering, and pathway enrichment analysis were performed using iDEP (Ge et al., 2018). Genes were ranked by their standard deviation across all samples, and the top 1,000 genes were used for hierarchical clustering analysis, and the top 2,000 genes were used for k-means clustering analysis (Ge et al., 2018). Gene set enrichment analysis was performed using GSEA software. Gene sets for murine CMP and GMP signatures were created by comparing gene expressions in bulk CMPs and GMPs using our RNA-seq data (Table S1). Our RNA-seq data are available in the Gene Expression Omnibus under accession number GSE166065. CD62L-low CMP and CD62L-low GMP in this dataset are described as CD62L-neg CMPs and CD62L-int GMPs in this article, respectively.

Statistical analysis

Significance of differences between two groups was assessed with unpaired two-tailed t tests, and three or more groups with one-way ANOVA. Differences were considered statistically significant

at a p value of less than 0.05. We used Prism 8 software for statistical analysis and graphical design.

Data and code availability

Our RNA-seq data are available in the Gene Expression Omnibus under accession number GSE166065.

SUPPLEMENTAL INFORMATION

Supplemental information can be found online at https://doi.org/10.1016/j.stemcr.2021.10.012.

AUTHOR CONTRIBUTIONS

Y.I. designed the research, performed experiments, analyzed data, and wrote the manuscript. F.N. designed the research and supervised experiments. Y.K. conceptualized and designed the research, supervised experiments, and wrote the manuscript. M.K. conceptualized and designed the research and supervised experiments.

CONFLICT OF INTERESTS

The authors declare no competing interests.

ACKNOWLEDGMENTS

CAG-EGFP mice were purchased from RIKEN BRC (Tsukuba, Japan).

Received: April 11, 2021
Revised: October 19, 2021
Accepted: October 20, 2021
Published: November 18, 2021

REFERENCES

Akashi, K., Traver, D., Miyamoto, T., and Weissman, I.L. (2000). A clonogenic common myeloid progenitor that gives rise to all myeloid lineages. Nature *404*, 193–197.

Buenrostro, J.D., Corces, M.R., Lareau, C.A., Wu, B., Schep, A.N., Aryee, M.J., Majeti, R., Chang, H.Y., and Greenleaf, W.J. (2018). Integrated single-cell analysis maps the continuous regulatory landscape of human hematopoietic differentiation. Cell *173*, 1535–1548.e16.

Cantor, A.B., and Orkin, S.H. (2002). Transcriptional regulation of erythropoiesis: an affair involving multiple partners. Oncogene *21*, 3368–3376.

Cho, S., and Spangrude, G.J. (2011). Enrichment of functionally distinct mouse hematopoietic progenitor cell populations using CD62L. J. Immunol. *187*, 5203–5210.

Choi, J., Baldwin, T.M., Wong, M., Bolden, J.E., Fairfax, K.A., Lucas, E.C., Cole, R., Biben, C., Morgan, C., Ramsay, K.A., et al. (2019). Haemopedia RNA-seq: a database of gene expression during haematopoiesis in mice and humans. Nucleic Acids Res. *47*, D780–D785.

Dinh, H.Q., Eggert, T., Meyer, M.A., Zhu, Y.P., Olingy, C.E., Llewellyn, R., Wu, R., and Hedrick, C.C. (2020). Coexpression of CD71 and CD117 identifies an early unipotent neutrophil progenitor population in human bone marrow. Immunity *53*, 319–334.e6.

Dress, R.J., Dutertre, C.A., Giladi, A., Schlitzer, A., Low, I., Shadan, N.B., Tay, A., Lum, J., Kairi, M.F.B.M., Hwang, Y.Y., et al. (2019). Plasmacytoid dendritic cells develop from Ly6D+ lymphoid progenitors distinct from the myeloid lineage. Nat. Immunol. *20*, 852–864.

Drexler, H.G., Zaborski, M., and Quentmeier, H. (1997). Thrombopoietin supports the continuous growth of cytokine-dependent human leukemia cell lines. Leukemia *11*, 541–551.

Drissen, R., Thongjuea, S., Theilgaard-Mönch, K., and Nerlov, C. (2019). Identification of two distinct pathways of human myelopoiesis. Sci. Immunol. *4*, eaau7148.

Forejtnikovà, H., Vieillevoye, M., Zermati, Y., Lambert, M., Pellegrino, R.M., Guihard, S., Gaudry, M., Camaschella, C., Lacombe, C., Roetto, A., et al. (2010). Transferrin receptor 2 is a component of the erythropoietin receptor complex and is required for efficient erythropoiesis. Blood *116*, 5357–5367.

Ge, S.X., Son, E.W., and Yao, R. (2018). iDEP: an integrated web application for differential expression and pathway analysis of RNA-Seq data. BMC Bioinformatics *19*, 1–24.

Ivetic, A., Green, H.L.H., and Hart, S.J. (2019). L-Selectin: a major regulator of leukocyte adhesion, migration and signaling. Front. Immunol. *10*, 1–22.

Jacobsen, S.E.W., and Nerlov, C. (2019). Haematopoiesis in the era of advanced single-cell technologies. Nat. Cell Biol. *21*, 2–8.

Kawamura, S., Onai, N., Miya, F., Sato, T., Tsunoda, T., Kurabayashi, K., Yotsumoto, S., Kuroda, S., Takenaka, K., Akashi, K., et al. (2017). Identification of a human clonogenic progenitor with strict monocyte differentiation potential: a counterpart of mouse cMoPs. Immunity *46*, 835–848.e4.

Kiatpakdee, B., Sato, K., Otsuka, Y., Arashiki, N., Chen, Y., Tsumita, T., Otsu, W., Yamamoto, A., Kawata, R., Yamazaki, J., et al. (2020). Cholesterol-binding protein TSPO2 coordinates maturation and proliferation of terminally differentiating erythroblasts. J. Biol. Chem. *295*, 8048–8063.

Kurotaki, D., Osato, N., Nishiyama, A., Yamamoto, M., Ban, T., Sato, H., Nakabayashi, J., Umehara, M., Miyake, N., Matsumoto, N., et al. (2013). Essential role of the IRF8-KLF4 transcription factor cascade in murine monocyte differentiation. Blood *121*, 1839–1849.

Kwok, I., Becht, E., Xia, Y., Ng, M., Teh, Y.C., Tan, L., Evrard, M., Li, J.L.Y., Tran, H.T.N., Tan, Y., et al. (2020). Combinatorial single-cell analyses of granulocyte-monocyte progenitor heterogeneity reveals an early uni-potent neutrophil progenitor. Immunity *53*, 303–318.

Laurenti, E., and Göttgens, B. (2018). From haematopoietic stem cells to complex differentiation landscapes. Nature *553*, 418–426.

Loughran, S.J., Haas, S., Wilkinson, A.C., Klein, A.M., and Brand, M. (2020). Lineage commitment of hematopoietic stem cells and progenitors: insights from recent single cell and lineage tracing technologies. Exp. Hematol. *88*, 1–6.

Manz, M.G., Miyamoto, T., Akashi, K., and Weissman, I.L. (2002). Prospective isolation of human clonogenic common myeloid progenitors. Proc. Natl. Acad. Sci. U S A *99*, 11872–11877.

Miyawaki, K., Iwasaki, H., Jiromaru, T., Kusumoto, H., Yurino, A., Sugio, T., Uehara, Y., Odawara, J., Daitoku, S., Kunisaki, Y., et al.

(2017). Identification of unipotent megakaryocyte progenitors in human hematopoiesis. Blood *129*, 3332–3343.

Mori, Y., Chen, J.Y., Pluvinage, J.V., Seita, J., and Weissman, I.L. (2015). Prospective isolation of human erythroid lineage-committed progenitors. Proc. Natl. Acad. Sci. U S A *112*, 2–7.

Nestorowa, S., Hamey, F.K., Pijuan Sala, B., Diamanti, E., Shepherd, M., Laurenti, E., Wilson, N.K., Kent, D.G., and Göttgens, B. (2016). A single-cell resolution map of mouse hematopoietic stem and progenitor cell differentiation. Blood *128*, e20–e31.

Nishikii, H., Kanazawa, Y., Umemoto, T., Goltsev, Y., Matsuzaki, Y., Matsushita, K., Yamato, M., Nolan, G.P., Negrin, R., and Chiba, S. (2015). Unipotent megakaryopoietic pathway bridging hematopoietic stem cells and mature megakaryocytes. Stem Cells *33*, 2196–2207.

Notta, F., Zandi, S., Takayama, N., Dobson, S., Gan, O.I., Wilson, G., Kaufmann, K.B., McLeod, J., Laurenti, E., Dunant, C.F., et al. (2016). Distinct routes of lineage development reshape the human blood hierarchy across ontogeny. Science *351*, aab2116.

Okabe, M., Ikawa, M., Kominami, K., Nakanishi, T., and Nishimune, Y. (1997). "Green mice" as a source of ubiquitous green cells. FEBS Lett. *407*, 313–319.

Olsson, A., Venkatasubramanian, M., Chaudhri, V.K., Aronow, B.J., Salomonis, N., Singh, H., and Grimes, H.L. (2016). Single-cell analysis of mixed-lineage states leading to a binary cell fate choice. Nature *537*, 698–702.

Osawa, M., Yamaguchi, T., Nakamura, Y., Kaneko, S., Onodera, M., Sawada, K.I., Jegalian, A., Wu, H., Nakauchi, H., and Iwama, A. (2002). Erythroid expansion mediated by the Gfi-1B zinc finger protein: role in normal hematopoiesis. Blood *100*, 2769–2777.

Paul, F., Arkin, Y., Giladi, A., Jaitin, D.A., Kenigsberg, E., Keren-Shaul, H., Winter, D., Lara-Astiaso, D., Gury, M., Weiner, A., et al. (2015). Transcriptional heterogeneity and lineage commitment in myeloid progenitors. Cell *163*, 1663–1677.

Perry, C., and Soreq, H. (2002). Transcriptional regulation of erythropoiesis fine tuning of combinatorial multi-domain elements. Eur. J. Biochem. *269*, 3607–3618.

Perry, S.S., Wang, H., Pierce, L.J., Yang, A.M., Tsai, S., and Spangrude, G.J. (2004). L-Selectin defines a bone marrow analog to the thymic early T-lineage progenitor. Blood *103*, 2990–2996.

Rapin, N., Bagger, F.O., Jendholm, J., Mora-Jensen, H., Krogh, A., Kohlmann, A., Thiede, C., Borregaard, N., Bullinger, L., Winther, O., et al. (2014). Comparing cancer vs normal gene expression profiles identifies new disease entities and common transcriptional programs in AML patients. Blood *123*, 894–904.

Song, S.H., Kim, A., Dale, R., and Dean, A. (2012). Ldb1 regulates carbonic anhydrase 1 during erythroid differentiation. Biochim. Biophys. Acta *1819*, 885–891.

Tak, T., Wijten, P., Heeres, M., Pickkers, P., Scholten, A., Heck, A.J.R., Vrisekoop, N., Leenen, L.P., Borghans, A.M., Tesselaar, K., et al. (2017). Human CD62L dim neutrophils identified as a separate subset by proteome profiling and in vivo pulse-chase labeling. Blood *129*, 3476–3485.

Velten, L., Haas, S.F., Raffel, S., Blaszkiewicz, S., Islam, S., Hennig, B.P., Hirche, C., Lutz, C., Buss, E.C., Nowak, D., et al. (2017). Human haematopoietic stem cell lineage commitment is a continuous process. Nat. Cell Biol. *19*, 271–281.

Weinreb, C., Rodriguez-Fraticelli, A., Camargo, F.D., and Klein, A.M. (2020). Lineage tracing on transcriptional landscapes links state to fate during diffrentiation. Science *367*, eaaw3381.

Yáñez, A., Ng, M.Y., Hassanzadeh-Kiabi, N., and Goodridge, H.S. (2015). IRF8 acts in lineage-committed rather than oligopotent progenitors to control neutrophil vs monocyte production. Blood *125*, 1452–1459.

Stem Cell Reports, Volume *16*

Supplemental Information

CD62L expression level determines the cell fate of myeloid progenitors

Yusuke Ito, Fumio Nakahara, Yuki Kagoya, and Mineo Kurokawa

Supplemental Figure 1 – CD62L expression reveals the heterogeneity of CMPs in mice and humans

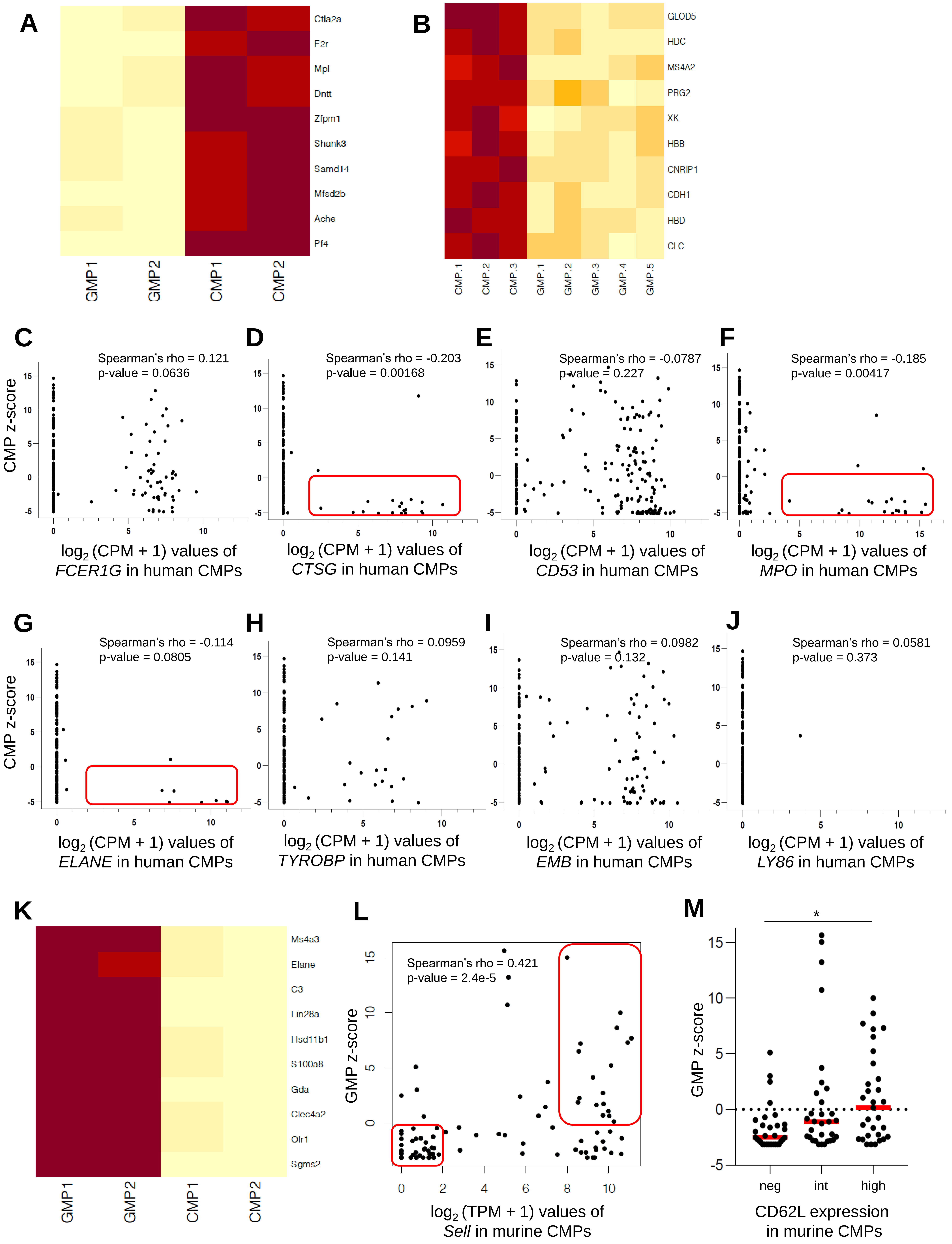

Supplemental Figure 2 – CD62L expression reveals the heterogeneity of CMPs *in vitro*

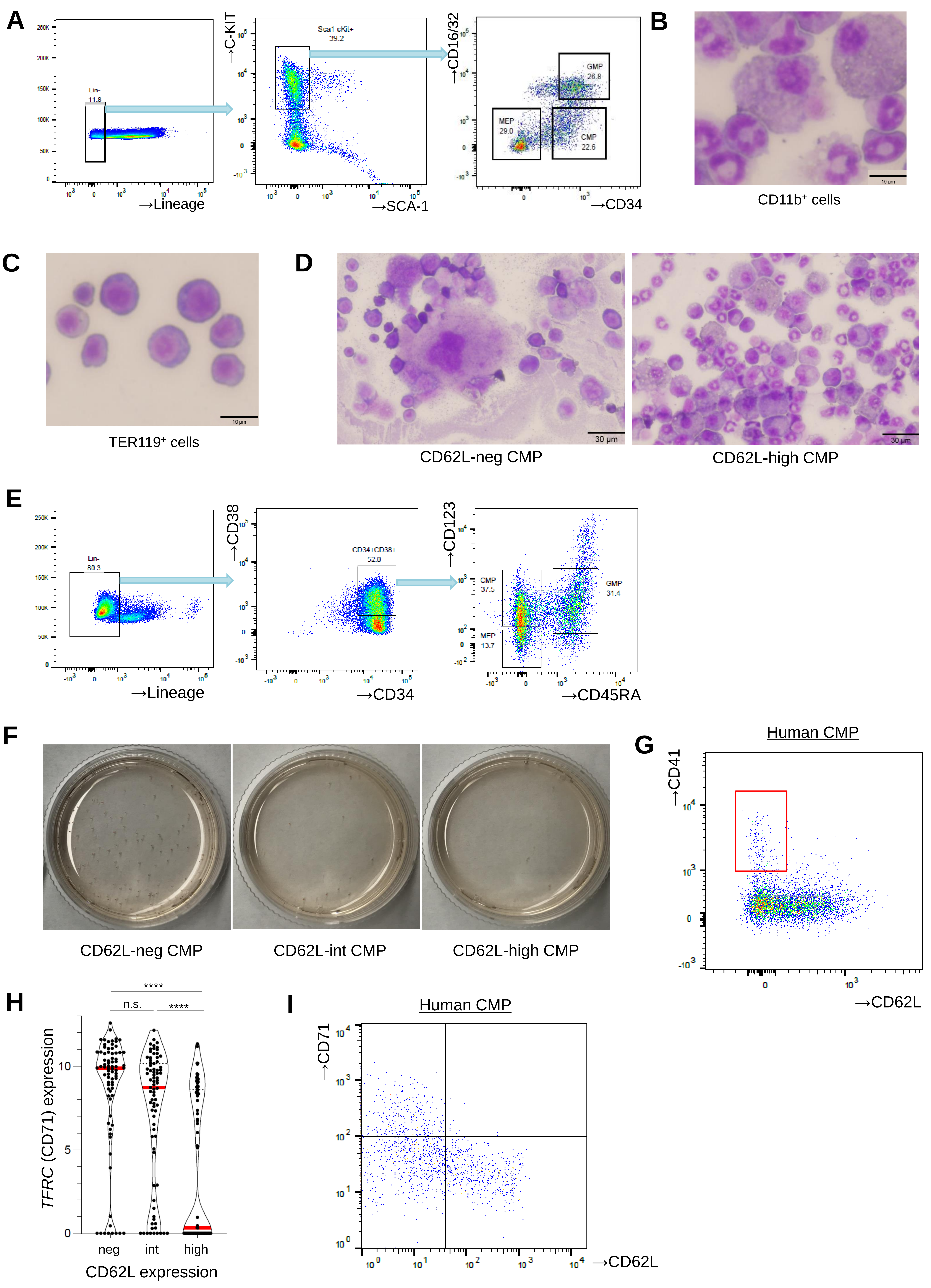

Supplemental Figure 3 – CD62L expression reveals the heterogeneity of CMPs *in vivo*

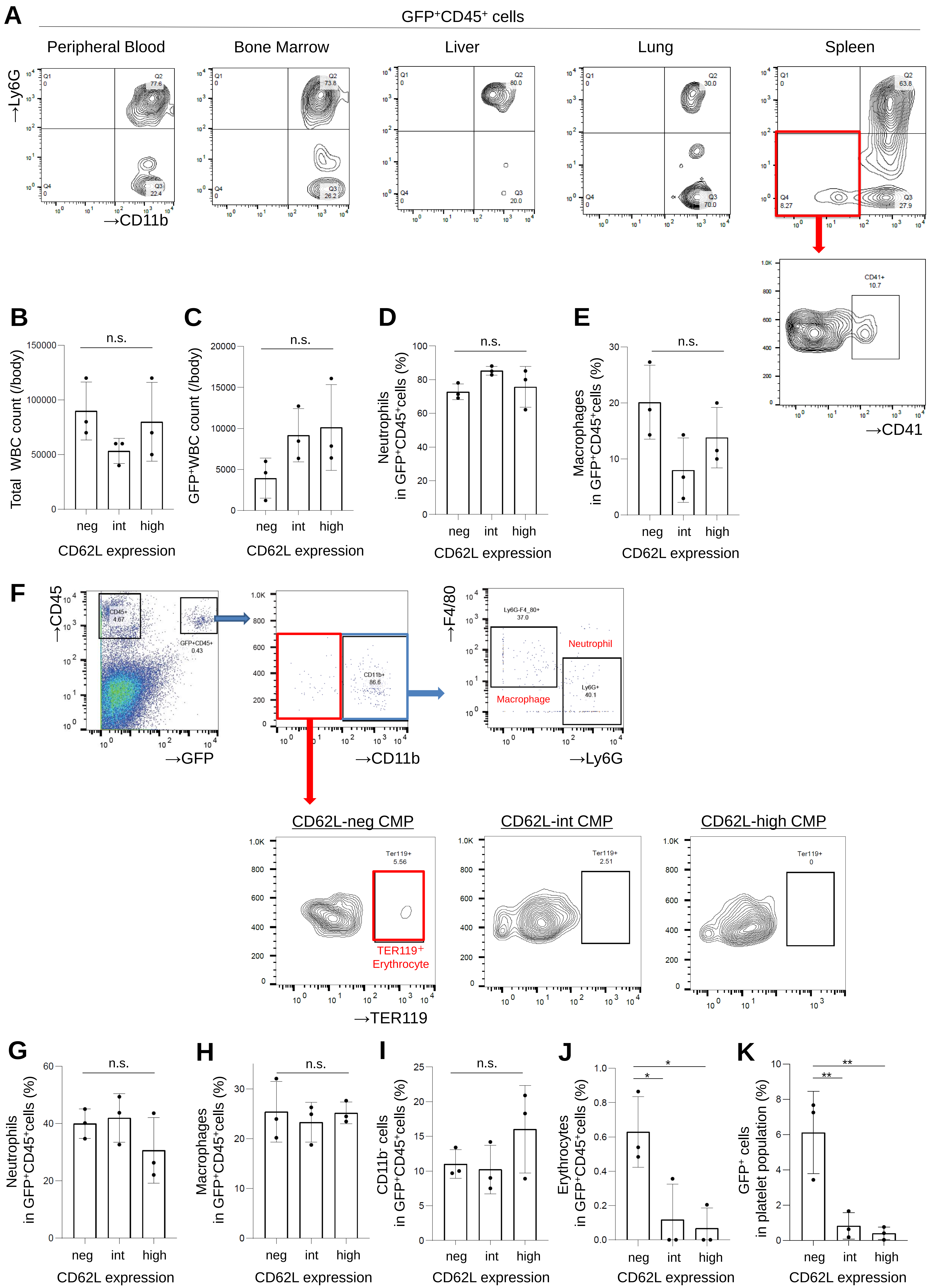

Supplemental Figure 4 – Gene expression profiles of murine CMPs

A

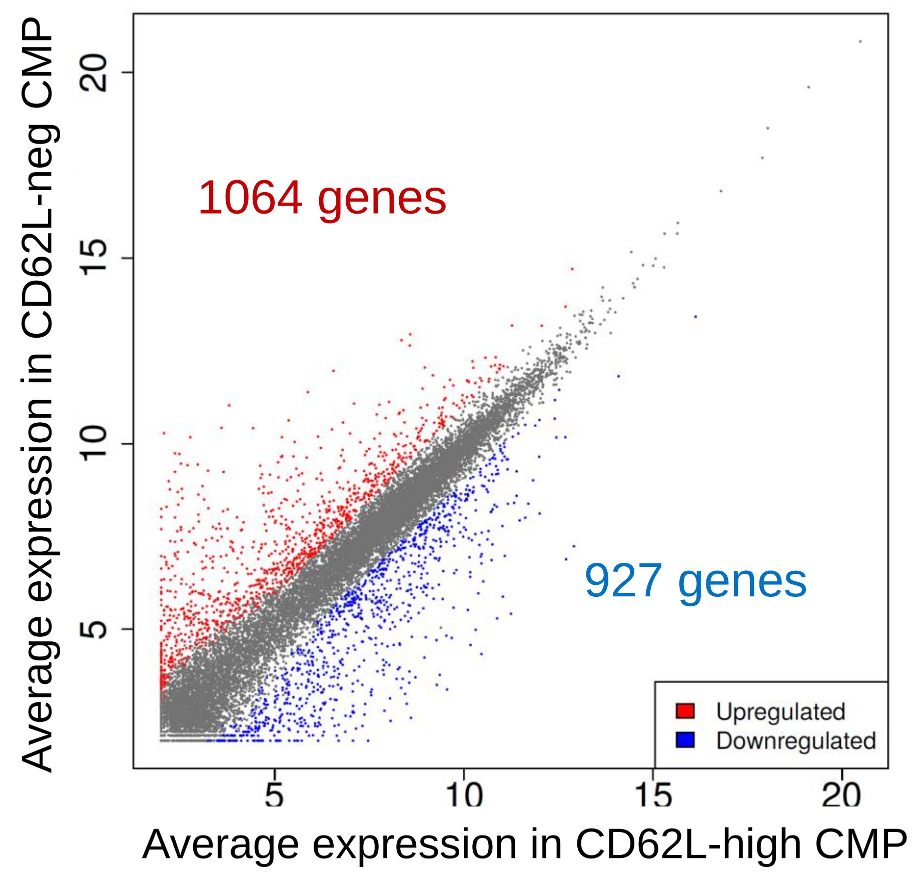

C

	p-value	Enriched Pathways in each cluster
A	1.2e-28	Immune system process
	1.2e-24	Defense response
	1.7e-24	Inflammatory response
	7.2e-24	Immune response
	1.6e-23	Response to external stimulus
	2.6e-21	Cytokine production
B	4.3e-18	Regulation of biological quality
	2.4e-17	Cell adhesion
	2.4e-17	Regulation of multicellular organismal process
	6.4e-16	System development
	7.4e-16	Regulation of response to stimulus
	2.6e-15	Blood coagulation
C	8.8e-08	Cell activation
	8.8e-08	Leukocyte activation
	1.4e-07	Positive regulation of immune system process
	2.5e-07	Cell surface receptor signaling pathway
	3.4e-07	Cell adhesion
	3.4e-07	Positive regulation of cell activation

B

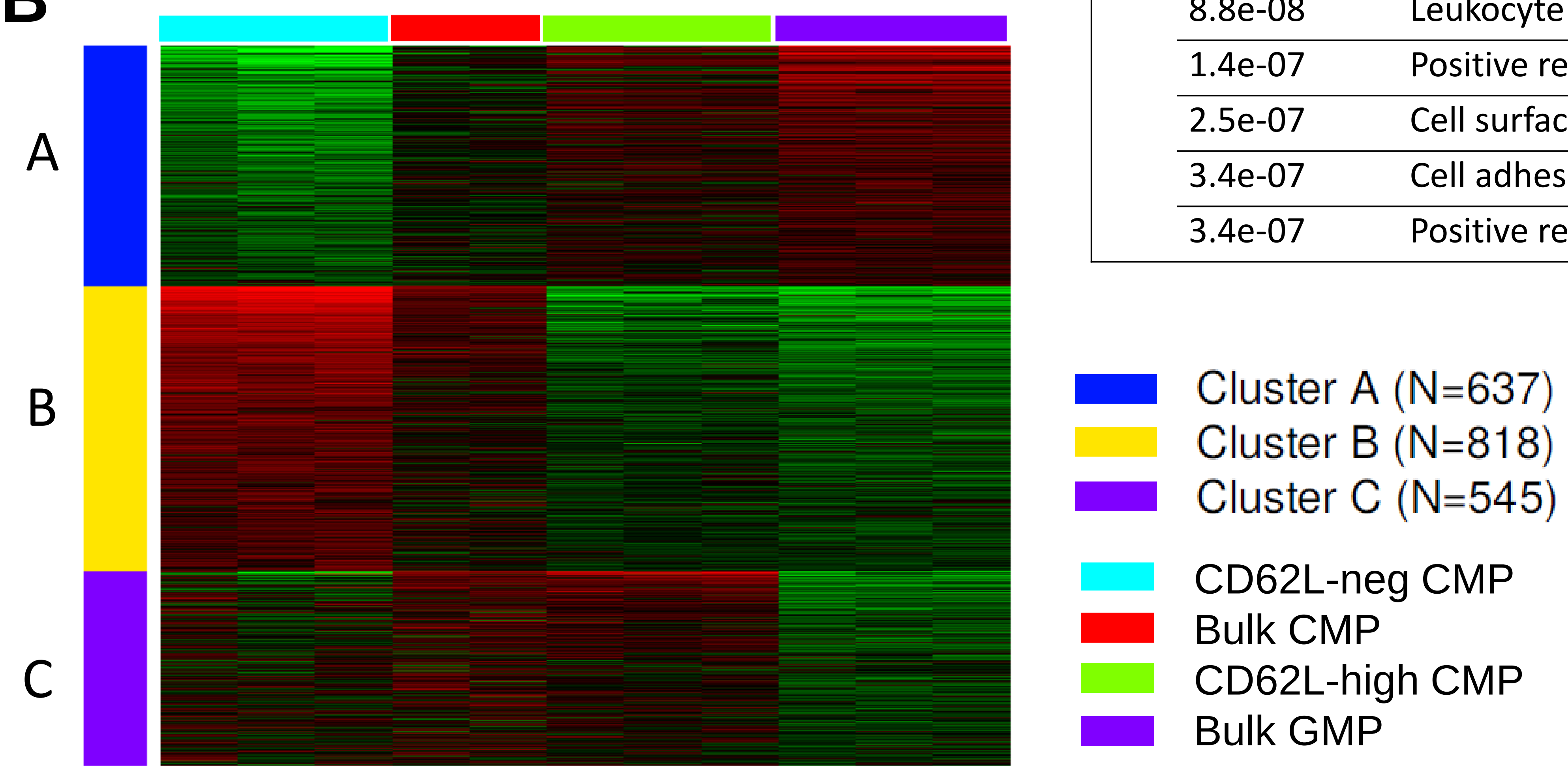

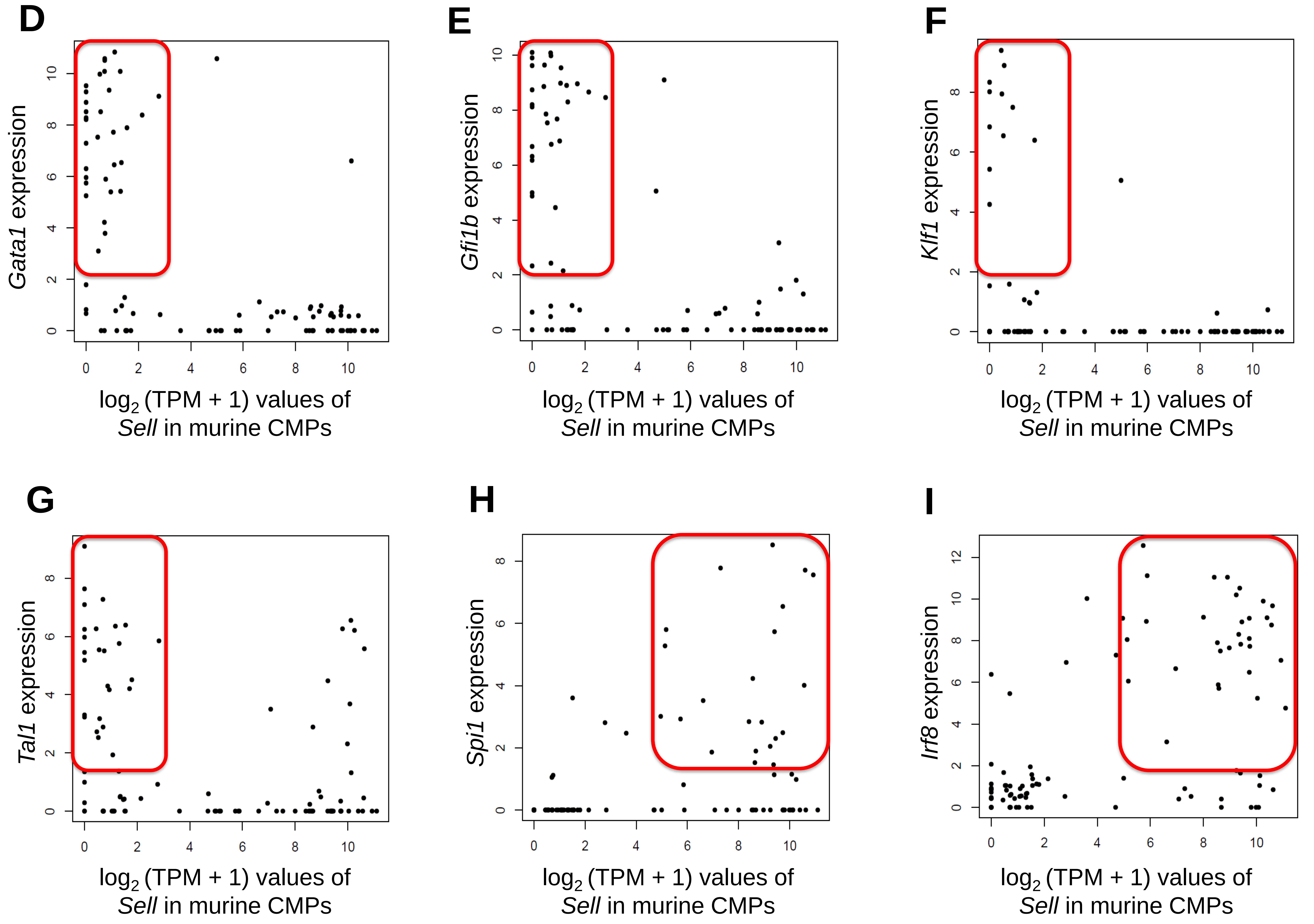

Supplemental Figure 5 – Single cell analyses confirm the heterogeneity of CMPs and GMPs

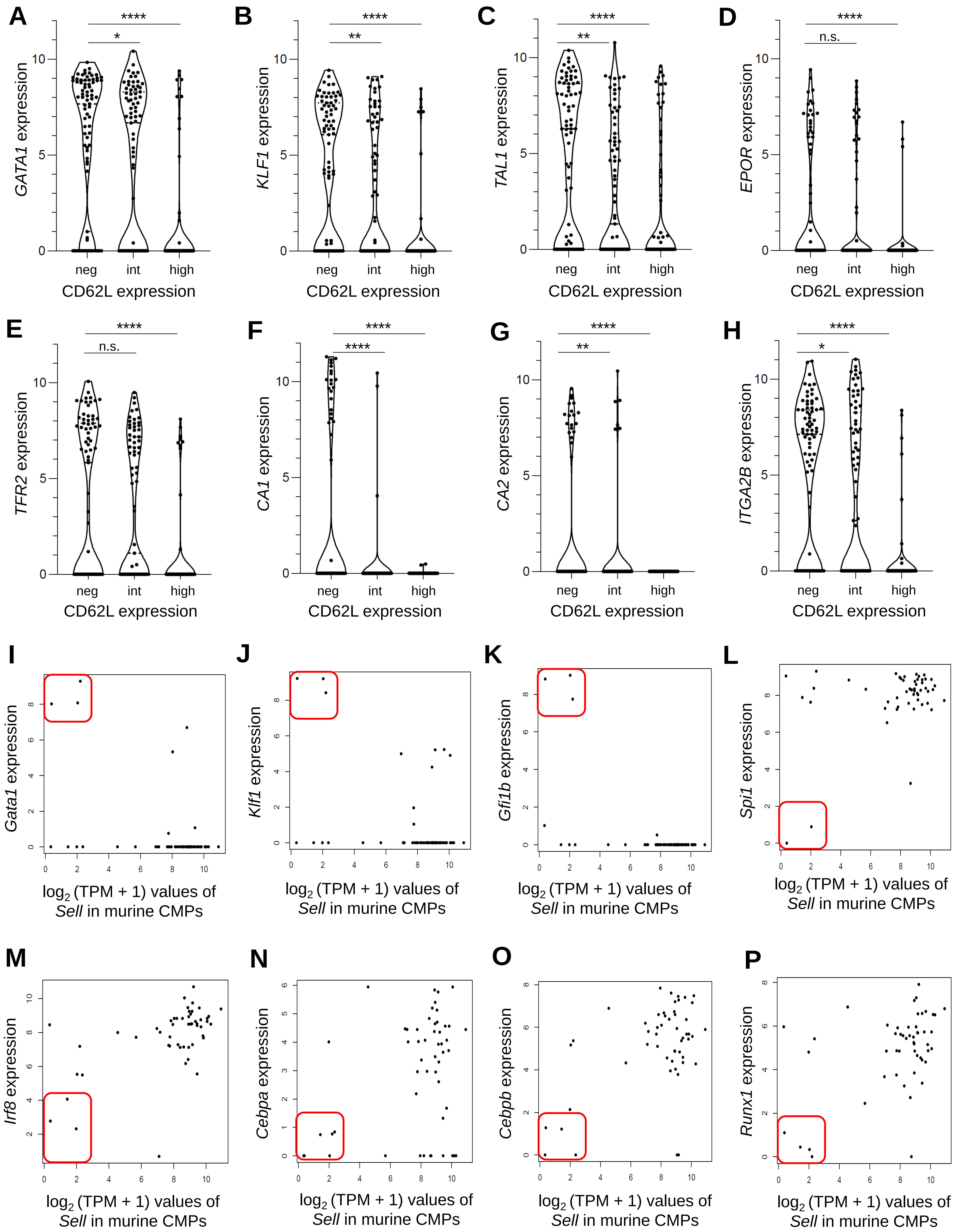

Supplemental Figure 6 – CD62L expression reveals the heterogeneity of GMPs in mice

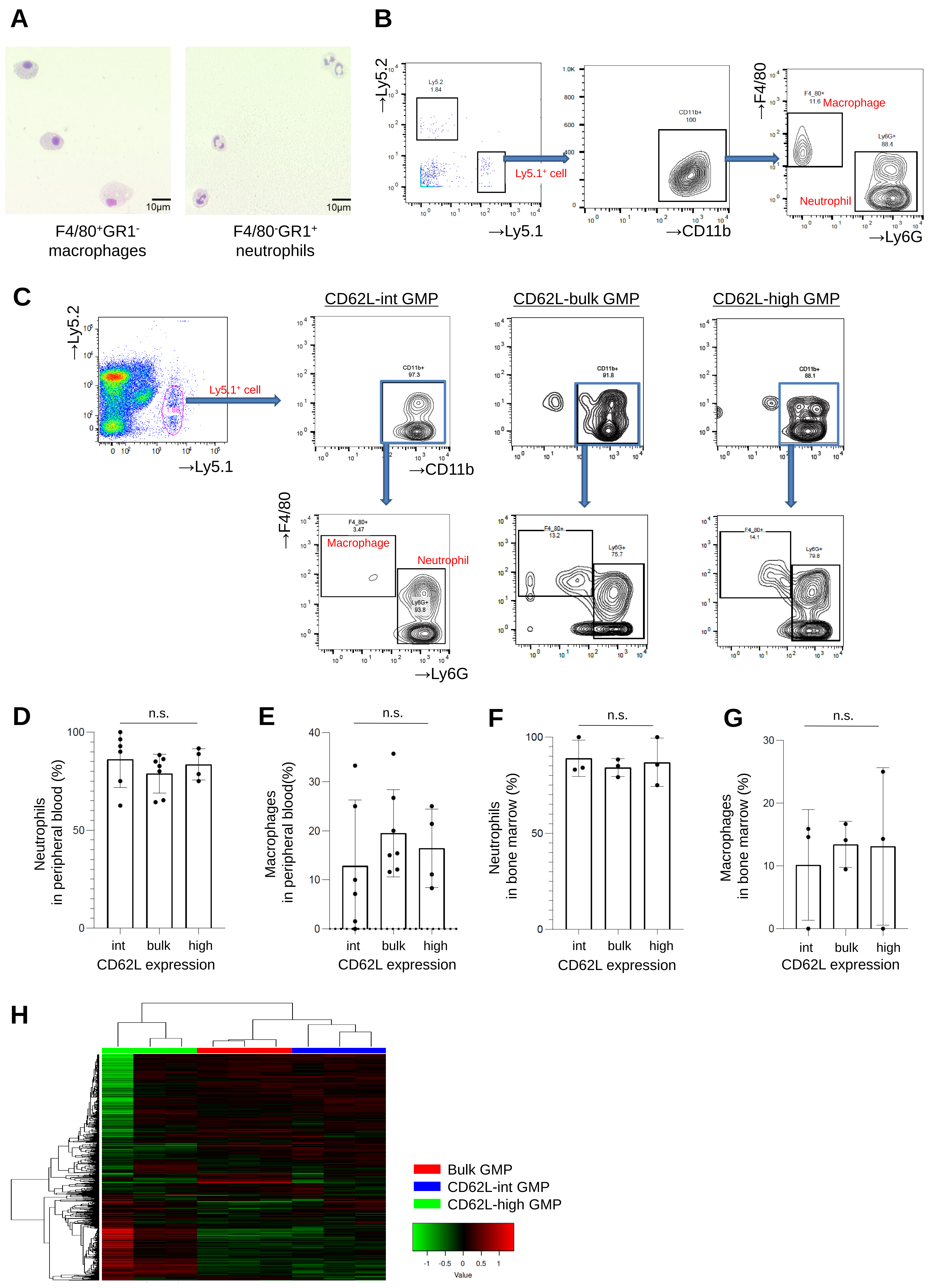

Supplemental Figure 7 – The summary of murine myeloid differentiation

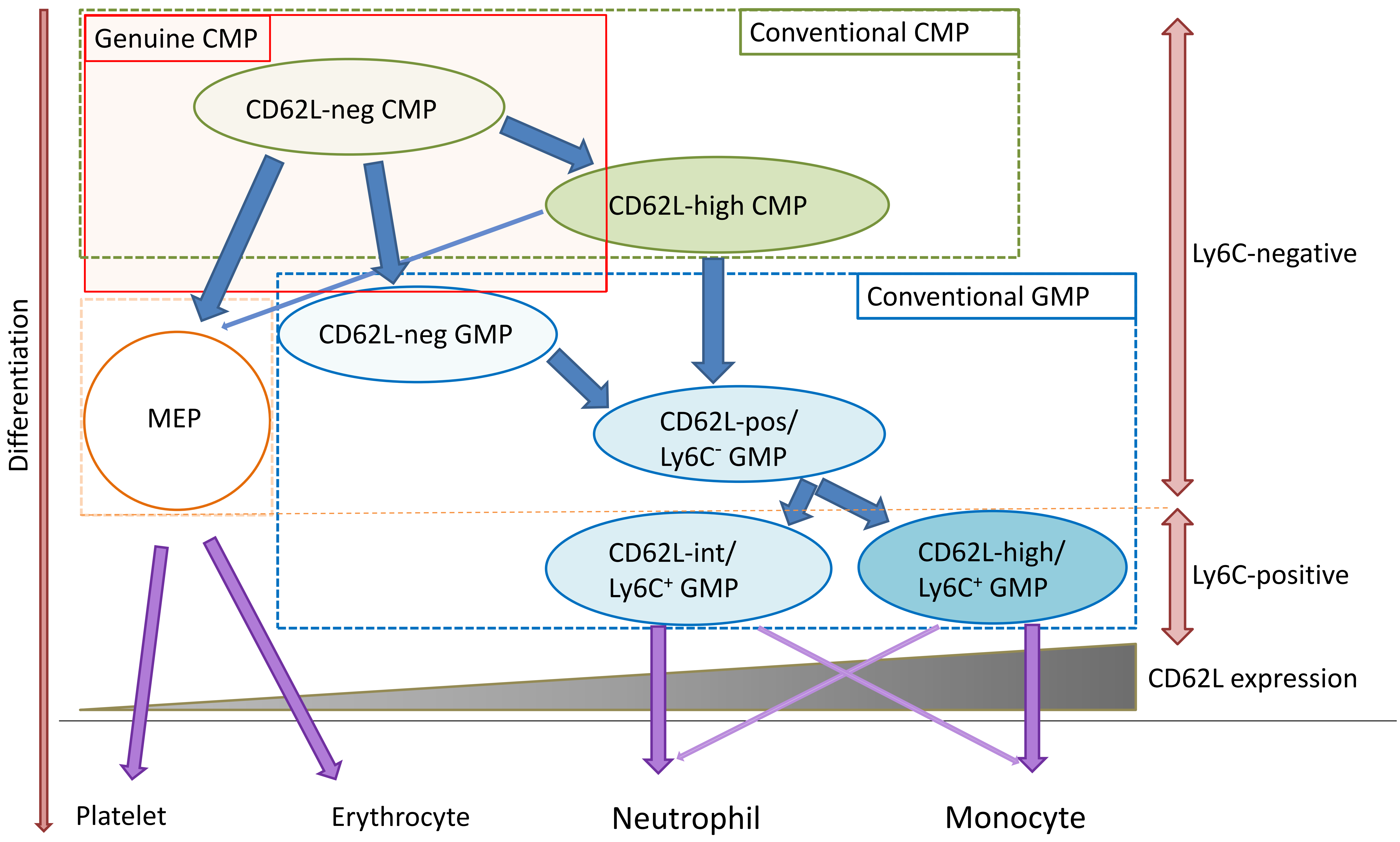

Supplemental Information

Supplemental Table legends

Table S1. Top 200 CMP and GMP signature gene sets in mice, related to Figure 4 and 6.

Murine CMP signature gene set and GMP signature gene set.

Supplemental Figure legends

Figure S1. CD62L expression reveals the heterogeneity of CMPs in mice and humans, related to Figure 1. (A and B) Heatmap of ten genes which were most highly expressed in CMPs compared with GMPs, using bulk RNA-seq data in mice (GSE116177) (A) and humans (GSE42519) (B). (C-J) Expression levels of each gene were plotted against the sum of CMP z-scores in individual cells in humans (C: *FCER1G*, D: *CTSG*, E: *CD53*, F: *MPO*, G: *ELANE*, H: *TYROBP*, I: *EMB*, J; *LY86*). X-axis shows $\log_2$ (CPM + 1) value of each gene and y-axis shows the sum of z-scores. (K) Heatmap of ten genes which were most highly expressed in murine GMPs compared with CMPs, using bulk RNA-seq data (GSE116177). (L) Expression levels of *Sell* were plotted against the sum of murine GMP z-scores in individual cells. X-axis shows $\log_2$ (TPM + 1) value of *Sell* and y-axis shows the sum of z-scores. (M) The plot

of the GMP z-scores divided by CD62L-neg, int, and high CMPs in mice. Statistical significance was calculated using one-way ANOVA. (*$p < 0.05$).

Figure S2. CD62L expression reveals the heterogeneity of CMPs *in vitro*, related to Figure 2. (A) Representative flow cytometry plots for sorting murine progenitors (CMP, GMP, and MEP) within mouse bone marrow cells. (B-D) Wright-Giemsa staining of cells after 7-day culture in semisolid medium. (B) $CD11b^+$ cells, (C) $TER119^+$ cells, (D) colonies derived from CD62L-neg and high GMPs. (E) Representative flow cytometry plots for sorting human progenitors (CMP, GMP, and MEP) within human $CD34^+$ bone marrow cells. (F) Macroscopic findings of colony-forming cell assay of human CMPs. CD62L-neg CMPs generate more red-colored BFU-Es than others. Experiments were performed three times. (G) Representative flow cytometry plots of CD41 and CD62L co-staining of human CMPs. Experiments were performed three times. (H) Violin plot of *TFRC*, a gene encoding CD71, in human CMPs using single cell RNA-seq data (GSE113046). CMPs were divided into CD62L-neg, int, and high CMPs, and Y-axis shows $\log_2$ (CPM + 1) of *TFRC* expression. Statistical significance was calculated using one-way ANOVA. ****$p < 0.0001$, n.s.: not significant. (I) Representative flow cytometry plots of CD71 and CD62L co-staining of human CMPs. Experiments were

performed three times.

Figure S3. CD62L expression reveals the heterogeneity of CMPs *in vivo*, related to Figure 3. (A) Representative flow cytometry plots of CD11b, Ly6G, and CD41 staining of GFP-positive hematopoietic cells collected from peripheral blood, bone marrow, liver, lung, and spleen in mice transplanted with CD62L-neg CMPs derived from GFP-mice. Experiments were performed three times. (B-E) The result of (B) total $CD45^+$ cell number, (C) GFP-positive cell number, and the proportion of (D) neutrophils and (E) macrophages in GFP-positive cells collected from bone marrow. Means ± S.D. of three independent experiments. Statistical significance was calculated using one-way ANOVA. (n.s.: not significant, $n = 3$). (F) Representative flow cytometry plots of neutrophils, macrophages, and erythrocytes in GFP-positive hematopoietic cells collected from splenic cells. Experiments were performed three times. (G-K) The result of the proportion of (G) neutrophils, (H) macrophages, (I) $CD11b^-$ cells, and (J) erythrocytes in GFP-positive hematopoietic cells and (K) the proportion of GFP-positive platelets in total platelets collected from spleen. Means ± S.D. of three independent experiments. Statistical significance was calculated using one-way ANOVA. (*$p < 0.05$, **$p < 0.01$, n.s.: not significant, $n = 3$).

Figure S4. Gene expression profiles of murine CMPs, related to Figure 4. (A) The scatter plot of differentially expressed genes between CD62L-neg CMPs and CD62L-high CMPs. Fold change > 2, FDR < 0.05. (B) The result of k-means clustering of bulk CMPs, CD62L-neg CMPs, CD62L-high CMPs, and bulk GMPs divided into cluster A, B, and C. (C) The result of pathway enrichment analysis of each cluster, showing top 6 significantly enriched pathways. (D-I) The relationship between several transcription factors and *Sell* expression in murine CMPs at single cell level (GSE70236). X-axis shows $\log_2$ (TPM + 1) of *Sell* and y-axis shows $\log_2$ (TPM + 1) of each transcription factor (D: *Gata1*, E: *Gfi1b*, F: *Klf1*, G: *Tal1*, H: *Spi1*, I: *Irf8*).

Figure S5. Single cell analyses confirm the heterogeneity of CMPs and GMPs, related to Figure 4 and 6. (A-H) Violin plots of several genes in human CMPs using single cell RNA-seq data (GSE113046). CMPs were divided into CD62L-neg, int, and high CMPs, and Y-axis shows $\log_2$ (CPM + 1) of each gene (A: *GATA1*, B: *KLF1*, C: *TAL1*, D: *EPOR*, E: *TFR2*, F: *CA1*, G: *CA2*, H: *ITGA2B* (CD41)). Statistical significance was calculated using one-way ANOVA. $^*p < 0.05$, $^{**}p < 0.01$, $^{****}p < 0.0001$, n.s.: not significant. (I-P) The relationship between several transcription factors

and CD62L expression in murine GMPs at single cell level (GSE130966). X-axis shows $\log_2$ (TPM + 1) of *Sell* and y-axis shows each transcription factor (I: *Gata1*, J: *Klf1*, K: *Gfi1b*, L: *Spi1*, M: *Irf8*, N: *Cebpa*, O: *Cebpb*, P: *Runx1*).

Figure S6. CD62L expression reveals the heterogeneity of GMPs in mice, related to Figure 7. (A) May-Giemsa staining of F4/80$^+$ macrophages and GR-1$^+$ neutrophils after 7-day culture of murine GMPs. (B and C) Representative plots of neutrophils and macrophages in the Ly5.1-positive donor cell population in (B) peripheral blood and (C) spleen are shown. (D-G) The result of the proportion of (D) neutrophils and (E) macrophages in peripheral blood, and (F) neutrophils and (G) macrophages in bone marrow in Ly5.1-positive donor cell population. Means ± S.D. of three to six independent experiments. Statistical significance was calculated using one-way ANOVA. (n.s.: not significant). (H) The result of hierarchical clustering of CD62L-int GMPs, CD62L-high GMPs, and bulk GMPs.

Figure S7. The summary of murine myeloid differentiation

Proposed model of CMP and GMP differentiation in mice. CD62L-neg CMPs are genuine CMPs and located at the upper differentiation hierarchy, whereas CD62L-high

CMPs were highly skewed to GMP potential. CD62L-negative GMPs are the most immature subsets, and differentiate into CD62L-positive GMPs in one direction. Then, Ly6C expression becomes upregulated and Ly6C^{+}/CD62L-int GMPs are skewed to neutrophil differentiation, whereas Ly6C^{+}/CD62-high GMPs are skewed to monocyte differentiation.
